# The SocialAI school: a framework leveraging developmental psychology toward artificial socio-cultural agents

**DOI:** 10.3389/fnbot.2024.1396359

**Published:** 2024-10-09

**Authors:** Grgur Kovač, Rémy Portelas, Peter Ford Dominey, Pierre-Yves Oudeyer

**Affiliations:** ^1^Flowers Team, INRIA, Bordeaux, France; ^2^Ubisoft La Forge, Bordeaux, France; ^3^INSERM UMR1093-CAPS, Université Bourgogne, Dijon, France; ^4^Robot Cognition Laboratory, Institute Marey, Dijon, France

**Keywords:** social cognition, developmental psychology, reinforcement learning, large language models, Michael Tomasello

## Abstract

Developmental psychologists have long-established socio-cognitive abilities as fundamental to human intelligence and development. These abilities enable individuals to enter, learn from, and contribute to a surrounding culture. This drives the process of cumulative cultural evolution, which is responsible for humanity's most remarkable achievements. AI research on social interactive agents mostly concerns the *emergence* of culture in a multi-agent setting (often without a strong grounding in developmental psychology). We argue that AI research should be informed by psychology and study socio-cognitive abilities enabling to *enter* a culture as well. We draw inspiration from the work of Michael Tomasello and Jerome Bruner, who studied socio-cognitive development and emphasized the influence of a cultural environment on intelligence. We outline a broader set of concepts than those currently studied in AI to provide a foundation for research in artificial social intelligence. Those concepts include social cognition (joint attention, perspective taking), communication, social learning, formats, and scaffolding. To facilitate research in this domain, we present The SocialAI school—a tool that offers a customizable parameterized suite of procedurally generated environments. This tool simplifies experimentation with the introduced concepts. Additionally, these environments can be used both with multimodal RL agents, or with pure-text Large Language Models (LLMs) as interactive agents. Through a series of case studies, we demonstrate the versatility of the SocialAI school for studying both RL and LLM-based agents. Our motivation is to engage the AI community around social intelligence informed by developmental psychology, and to provide a user-friendly resource and tool for initial investigations in this direction. Refer to the project website for code and additional resources: https://sites.google.com/view/socialai-school.

## 1 Introduction

Our everyday life is immersed in a sociocultural world, which we navigate using a set of sophisticated socio-cognitive abilities. Although at first it might seem that this sociocultural world is just another downstream product of our cognition, decades of research in developmental psychology suggest the opposite. Our socio-cultural world, cultural knowledge, and our socio-cognitive abilities are the foundation of our development and both our social and asocial intelligence (Vygotsky and Cole, 1978; Bruner, 1990; Tomasello, 2019).

For Vygotsky, a main driver for “higher-level” cognition are socio-cultural interactions (Vygotsky and Cole, 1978). He argues that many high-level cognitive functions first appear at the social level and *then* develop at the individual level. This leap from interpersonal processes to intrapersonal processes is referred to as *internalization*. A typical example of this process is learning to count. Children first learn to count out loud, i.e. with language and social guidance, which is an interpersonal process. As the child improves, it will learn to count in its head, no longer requiring any external guidance: counting became internalized, and will be a first step toward other more complex forms of abstract thinking. Vygotsky's theories influenced multiple works within cognitive science (Clark, 1996; Hutchins, 1996), primatology (Tomasello, 1999) and the developmental robotics branch of AI (Billard and Dautenhahn, 1998; Brooks et al., 2002; Cangelosi et al., 2010; Mirolli and Parisi, 2011).

Another pillar of modern developmental psychology is Jerome Bruner. He, too, emphasized the importance of culture in human development. Bruner (1990) writes: “*it is culture, not biology, that shapes human life and the human mind, that gives meaning to action by situating its underlying intentional states in an interpretative system*.” Most importantly for this paper, he presents a pragmatic view studying how referencing, requesting and finally language develop through routinized social interactions (formats) in which those abilities are *necessary* to achieve various ends. He describes these interactions as scaffolded— the caretaker gradually helps less and demands more of the child to achieve those goals, and this bootstraps the child's development (Bruner, 1985).

Finally, Michael Tomasello's work (Tomasello, 1999, 2019, 2020) constitutes a representative and contemporary assessment of the nature and central importance of sociality in human cognition. He outlined core social abilities and motivations through theoretical and experimental studies with humans and apes. When combined with the relevant experience, those abilities enable us to enter, benefit from, and contribute to the human culture, i.e. they enable the cumulative cultural evolution (a powerful form of cultural transmission fostering the development and perpetuation of complex culture and knowledge) (Tomasello, 1999).

Given the key role social cognition plays in human cognition and cultural evolution, it is natural that the field of AI aims to model social intelligence. A socially competent AI could learn our culture and participate in its cultural evolution, i.e. improve our concepts, theories, inventions, and create new ones. A system capable of out-of-the-box thinking creative solutions and discovering new relevant problems must learn our values and how we see and understand the world (it must learn our culture). We do not claim that The SocialAI is sufficient to reach that far and complex goal. We only propose that being informed by the concepts discussed in this paper is useful, and we present SocialAI as a tool which could be used to start investigating such questions in more details. Enriching AI with those skills also has numerous practical implications. Socially competent robots, capable of social learning, would be much easier to deploy and adapt to novel tasks and tools. For example, performing collaborative tasks with a robotic learner able to detect, learn and reuse context-dependent sets of communicative gestures/utterances could be easily integrated into human teams, without requiring humans to adopt new conventions. Furthermore, robots capable of learning human values and moral norms will be capable of performing tasks in the constraints defined by those values.

To further clarify the motivation of this work, we present an analogy with the skill of multiple object tracking (MOT). The MOT ability is an object of study under the umbrella of perception in developmental psychology. This ability enables humans, for instance, to drive a car. In AI, the ability of MOT was adopted as a research goal. An algorithm with this ability could at one point be used in applications involving high-level perception such as autonomous driving. However, it remains possible that autonomous driving could be solved without MOT as an intermediary step. Furthermore, the implementetion of a MOT capable system does not need to be similar to a human MOT system. Similarly, socially recursive inferences are an object of study under the umbrella of social cognition in developmental psychology. This ability enables humans, for instance, to play pictionary. We therefore arge that it should be adopted as a research goal, even though it remains possible that a pictionary playing system could be created without this intermediary step. Like for MOT, the implementation of such a system does not need to be similar to a human system for social inferences.

AI research on interactive agents is often focused on navigation and object manipulation problems, excised of any social dimension (Mnih et al., 2015; Lillicrap et al., 2015). The study of sociality is mostly studied in Multi-Agent settings, where the main focus is often on the *emergence* of culture (often with only a weak grounding in developmental psychology) (Jaques et al., 2019; Baker et al., 2019). While we believe that those directions are both interesting and important, in this work we focus on *entering* an already existing complex culture. And we argue that it can be beneficial to be informed by developmental psychology theories.

Cognitive science has inspired many works in social cognition in AI both in disembodied and embodied settings. In a disembodied setting, Machine learning models have been evaluated on their capacity to predict agent actions in theory of mind experiments. Rabinowitz et al. (2018) and more general social perception assessments (Netanyahu et al., 2021). In a virtual embodied setting, Jaques et al. (2019) implemented a model of social influence to foster coordination of MARL agents. Wu et al. (2021) study theory of mind in the context of collaboration and present the Bayesian Delegation algorithm to infer the intentions of others. Finally, in the real-world embodied setting, Cangelosi and Schlesinger (2014) discuss how to leverage knowledge from the cognitive development of human babies into embodied robots. Vollmer et al. (2016) argue that restricted predefined (not learned) interaction protocols (pragmatic frames) are usually used in the field of Human-Robot Interaction, and suggest studying a broader set of social situations. Our work following this rich tradition of levering insight from cognitive science and developmental psychology with focus on virtual embodied agents.

In the rapidly emerging field of Large Language models, social cognition research consists of proof-of-concept simulations (Park et al., 2023) and systematic benchmarks. Two most notable benchmarks are SiQA (Sap et al., 2019), which evaluates social common sense reasoning (without grounding in psychology), and ToMi (Le et al., 2019) which presents false-belief querries (false-belief representing only a small subset of social-intelligence in general). We believe this relevant and interesting work can be further enriched by an overview of different aspects of social intelligence and interactive settings presented in this work.

Following the theories of Michael Tomasello and Jerome Bruner, this work identifies a richer set of socio-cognitive skills than those currently considered in most of the AI research. More precisely, we focus on three key aspects of social cognition as identified by Tomasello: (1) social cognition: the ability to infer what others see and to engage in joint attention, (2) communication: the development of referential communication through pointing and the beginning of conventionalized communication through language, and (3) cultural learning: the use of imitation and role reversal imitation in social learning. We also outline two concepts from Jerome Bruner's work: formats and scaffolding. Formats refer to the way in which social interactions are structured, and scaffolding refers to the temporary support provided by a caretaker to help a learner achieve a task that would be otherwise too difficult.

**Figure 1:**
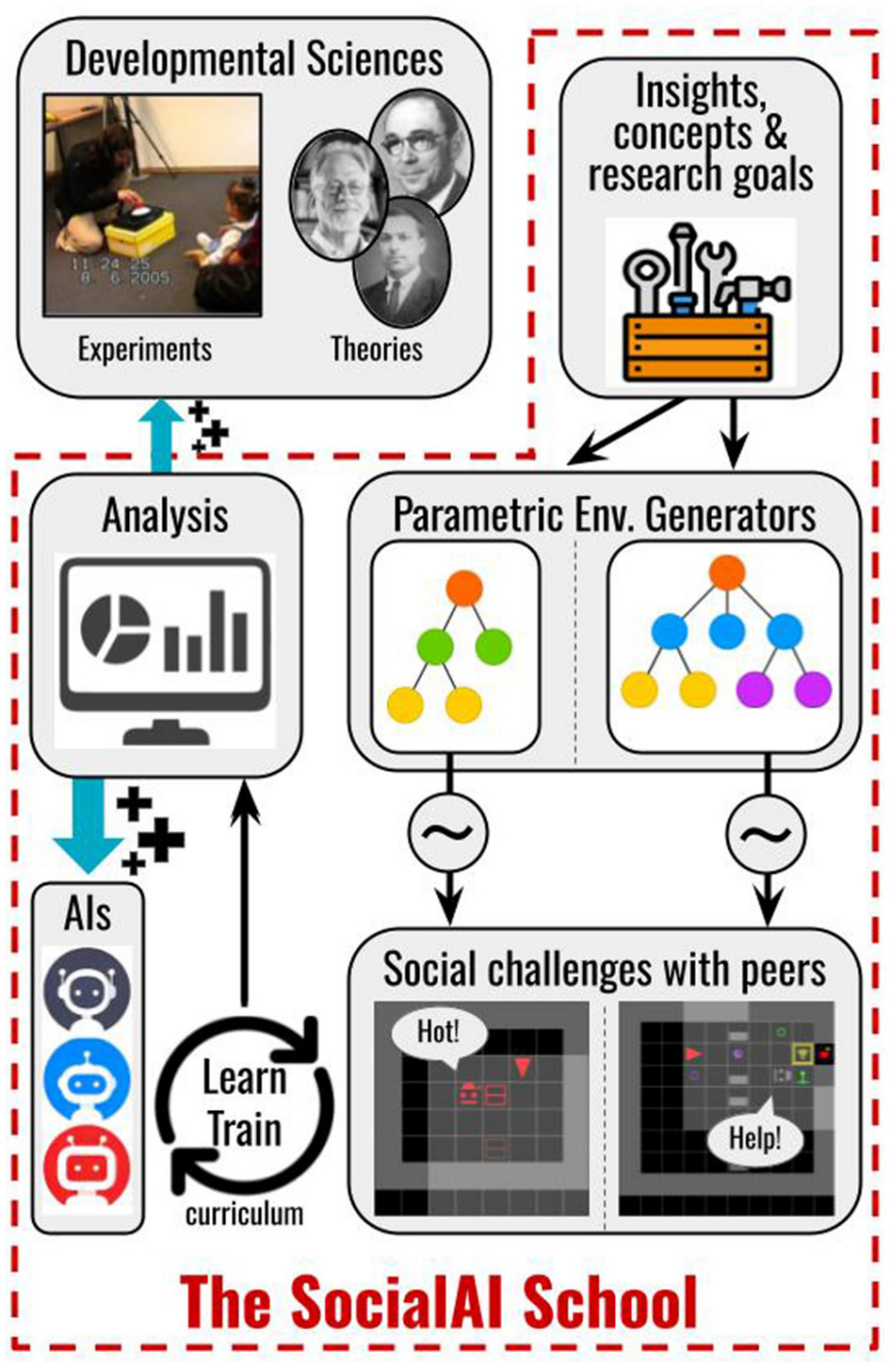
The SocialAI School contains technical and conceptual tools simplifying research of socially proficient AI agents. Developmental sciences give inspiration to the SocialAI School in the form of terminology and research goals, based on which we created a parametric environment generator. The user defines the parameters defining the sampling of environments (~). This enables the analysis and experiments in the form of training, giving insight into for building better AI (+) and even for developmental sciences (+).

Based on this set of target abilities, we construct the SocialAI school (shown in Figure 1), a tool [based on MiniGrid (Chevalier-Boisvert et al., 2018)] which enables the construction of social environments whose diverse grid-world scenarios affords rich yet tractable research around social competence acquisition. Considered social scenarios are organized according to the key cognitive science experiments used to study the social cognition in children by highlighting core developmental steps.

**Figure 2:**
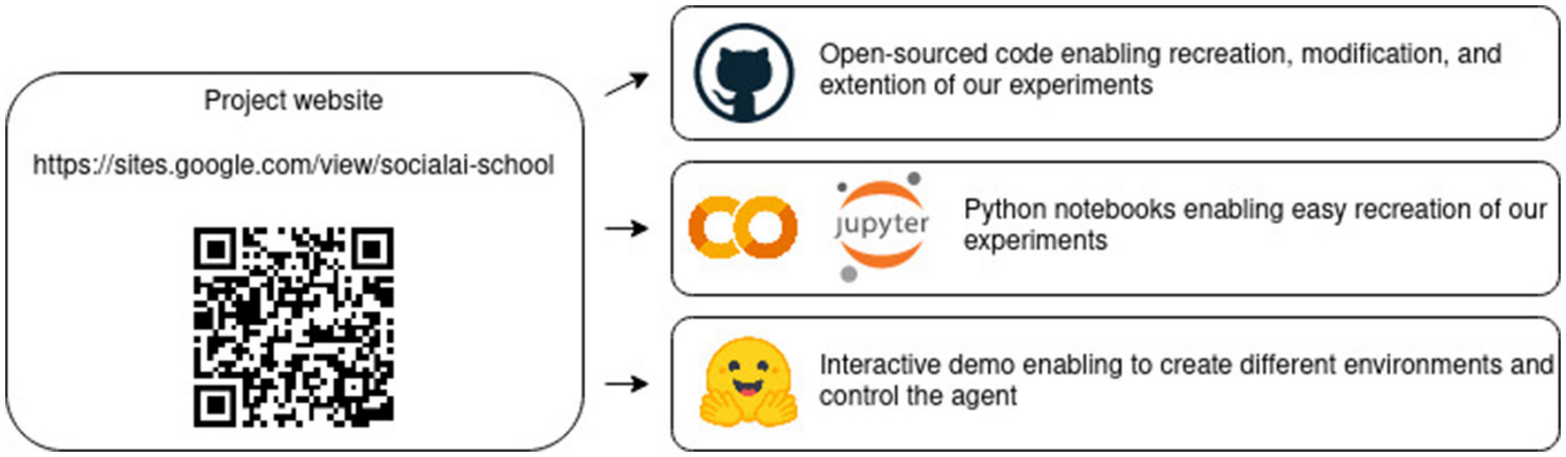
The SocialAI school is accompanied by additional resources available at the project's website.

We do not claim that the SocialAI school is sufficient to construct a socially competent agent as this is a very far-reaching and complex goal. However, we believe that in aiming for this goal, concepts from developmental psychology can serve as signposts for AI—give directions and enable us to define short term goals. Given that the outlined skills are at the very core of human social and cognitive competences, artificial agents aimed at participating in and learning from social interactions with humans are likely to require the same core competences. We present the SocialAI school merely as a first step toward this goal. The SocialAI school can be easily modified and extended. The code is open-sourced and accompanied by additional resources (see Figure 2).

In our experiments, we aim to show the versatility of the experiments which could be conducted with the SocialAI school. We present experiments regarding the following questions: generalization of social inferences (the pointing gesture) to new contexts, recreating an experiment from cognitive science (to study the knowledge transfer during role reversal), and the impact of a scaffolded environment on the agent's learning. To show the diversity of agents which can be used, we conduct those experiments with RL agents, and present an additional case study with LLMs as interactive agents. In the Supplementary material, we explore many more questions such as linguistic inferences, joint attention, and imitation. We hope to encourage future work extending and building on these first experiments to study various questions regarding social competence. For example, new socio-cultural scenarios, architectures, training regimes, and so on.

We outline the following main contributions of this work:

- An introduction to Michael Tomasello's and Jerome Bruner's theories on child development and core socio-cognitive abilities.
- An outline of a set of core socio-cognitive abilities important for current AI research (abilities that enable to *enter* a culture).
- The SocialAI school: a tool including a customizable procedural generation suite of social environments aiming to simplify studies of socio-cognitive abilities of AI agents.
- Examples of case studies demonstrating how SocialAI can be used to study various questions regarding socio-cognitive abilities in AI.

## 2 Cognitive science background

This section introduces Michael Tomasello's and Jerome Bruner's theories and concepts.

### 2.1 The shared intentionality theory (Michael Tomasello)

Humans are born into a culture filled with cultural artifacts, symbols and institutions like language, social norms, tool industries, or even governments (Richerson and Boyd, 2006; Tomasello, 2019). These artifacts are a product of a series of modifications over many generations. Tomasello calls this *cumulative cultural evolution*, and argues that it is behind our most impressive achievements (Tomasello, 1999).

Cumulative cultural evolution is grounded in our socio-cognitive abilities (e.g. social cognition, cultural learning, communication), which enable us to learn, improve, and teach our culture (Tomasello, 2019), i.e. *enter* a culture. Cultural artifacts inherited through this process become the core of our cognition. An example of this is language, which influences our cognition in many ways. For example, it defines how we categorize and construe the world, and enables a powerful form of social learning : instructed learning (Tomasello, 1999). This makes socio-cognitive abilities crucial, as their early development bootstraps both our social and asocial cognition (Herrmann et al., 2007).

Tomasello's *Shared intentionality theory* argues that human socio-cognitive abilities, such as communication and social learning, are transformed by two developmental steps : the emergence of *Joint intentionality* at around 9 months of age (the 9-month revolution), and the emergence of *Collective intentionality* at around 3 years of age (the objective/normative turn) (Tomasello, 2019).

**Joint intentionality** emerges at around 9 months of age (Tomasello, 2019). It enables children to form a *joint agent* (a dyadic “we”)—they understand that they work with a partner toward the same joint goal. Children begin to view dyadic social interactions through a “*dual-level structure”*: a joint agent “we” on one level, and a personal “I” on another, i.e. we both understand that we both have separate roles (“I”), and that we work together toward the same joint goal (“we”). This enables human children to take the perspective of others, which can also be done recursively (they are not only both attending to the same goal, they are also both attending to the partner's attention to the goal, and they both know that they both are doing so).

**Collective intentionality** emerges at around 3 years of age (Tomasello, 2019). It enables children to form a cultural *group-minded “we,”* which in comparison with a dyadic “we” represents an identity for a group. For example, a child might enforce a social norm because “this is how *we*, in this culture, do things.” Consequently, children begin to participate in conventions and norms, and to view things from the “objective” perspective.

These two developmental steps transform countless abilities, motivations, and behaviors. For the purpose of this paper, we focus on the following three developmental pathways: social cognition (Section 2.1.1), communication (Section 2.1.2), and social learning (Section 2.1.3), as we consider them the most relevant for AI at the moment.

#### 2.1.1 Social cognition

In this section, we discuss the development of the ability to coordinate perspectives and view things from the *objective perspective* (a perspective independent from any individual) (Tomasello, 2019). The starting point is the ability to **infer what another sees or knows**. The earliest example of this is gaze following of six-month-olds (D'Entremont et al., 1997). Here, only one perspective is processed at the time. **Joint attention (JA)** emerges at around 9 months of age. Tomasello (2019) defines JA as consisting of two elements: *triangulation* (two participants attending to the same referent) and *recursiveness* (both participants being recursively aware that they are both sharing attention). JA is characterized by the dual-level structure of shared attention (on one level) and individual perspectives (on another level). Consequently, children start to align and exchange perspectives. Once children reach a sufficient level of linguistic competence, they start sharing attention to mental content in the form of linguistic discourse (at two to three years of age). The presence of conflicting perspectives in linguistic discourse (e.g. a disagreement about where some object is located) pushes children to resolve those conflicts, which they do by forming the “objective” perspective, and **coordinating other perspectives** with it.

#### 2.1.2 Communication

Communication starts with **imperative gestures for self-serving purposes**. An example of such a gesture is the child pulling the adult's hand, requesting them to pick them up. This gesture always has the same imperative meaning, and it never refers to an external object. The 9-month revolution brings forth **referential communication**—children start to communicate triadically to external referents through pointing and pantomiming. The pointing gesture is a powerful way of communicating, as the *same* gesture can be used to express different meanings in different contexts (provided that the observer can infer that meaning). The ability to infer this meaning is based on the emerging abilities of joint intentionality. Those of joint attention and, most notably, of *socially recursive inferences*—to interpret a pointing gesture, we make a recursive inference of what “*you* intend for *me* to think.” For example, if we are looking for a ball together, and you point to a cupboard behind me. I should infer that you are drawing my attention to the cupboard to communicate that I should look for the ball in the cupboard. The next step is the appearance of **conventionalized linguistic communication**. The underlying principle stays the same: reference to an external entity combined with inferring the meaning through recursive inferences. The difference is that, now, the meaning depends on the conventional means (e.g. words and phrases) as well as the context. Tomasello argues that, at first, children don't understand language as conventional, and they use it as any other tool. The understanding of language as conventional follows the emergence of collective intentionality after the third birthday. This enables a myriad of different language uses, such as discourse or pedagogy.

#### 2.1.3 Cultural learning

Human culture is characterized by a powerful form of cultural transmission called cumulative cultural evolution—inventions quickly spread and are improved by following generations (Tomasello, 1999). These inventions spread at such a pace that they are rarely forgotten or lost. This is referred to as the *ratchet* effect (Tomasello et al., 1993)—inventions are iteratively improved without *slippage* back. This effect is enabled by human social learning abilities (ex. imitation, instructed learning), and motivations (to learn from others, but also to affiliate and conform). The earliest form of cultural learning is the **mimicking of facial expressions** [observed even in neonates (Meltzoff and Moore, 1997)]. Over the course of the first year, children begin to **imitate other's actions and goals**, and then, they begin doing so in ways which demonstrate their understanding of other's as intentional agents (Meltzoff, 1995). For example, in the failed-attempt paradigm children imitate a goal that the adult attempted, but failed, to reach. Joint intentionality brings forth a new form of cultural learning called **role reversal imitation**. Children can reverse the roles of a collaborative activity by learning about the partners role only from playing their own. For example, children respond to an adult tickling their arm, by tickling the adult's arm (instead of its own) (Carpenter et al., 2005). This is enabled by the dual-level structure of joint intentionality through which children understand, at the same time, the joint goal of a dyadic interaction on one level, and the individuals' separate roles on another. The next big step in the development of cultural learning is learning from instructions—**instructed learning** (following the emergence of collective intentionality). It is based on the adults' motivation to teach children as well as on the children's ability to understand and learn from linguistic instructions. Children understand knowledge acquired through instructions as objective truth, and generalize it much better than knowledge acquired by other means (Butler and Tomasello, 2016). In this way we acquire the most complex knowledge and skills such as reading or algebra.

### 2.2 Scaffolding and formats in Jerome Bruner's theory

This work is also influenced by Jerome Bruner's theories, especially regarding the concepts of scaffolding (Wood et al., 1976) and formats (Bruner, 1985), which were recently reintroduced to AI as pragmatic frames (Vollmer et al., 2016).

**Formats (Pragmatic frames)** (Bruner, 1985) simplify learning by providing a stable structure to social interactions. They are regular patterns characterizing the unfolding of possible social interactions (equivalent to an interaction protocol or a grammar of social interactions). Formats consist of a deep structure (the static part) and a surface structure (the varying realizations managed by some rules). An example of a format is the common peek-a-boo game. The deep structure refers to the appearance and the reappearance of an object. The surface structure can be realized in different ways. For example, one might hide an object using a cloth, or hands; one might hide his face or a toy; one might do shorter or longer pauses before making the object reappear. We understand social interactions through such formats, and our social interactions are based on our ability to learn, negotiate, and use them.

Another relevant concept is **scaffolding** (Wood et al., 1976) [similar to Vygotsky's zone of proximal development (Vygotsky and Cole, 1978)]. Scaffolding is a process through which an adult bootstraps the child's learning. The adult controls aspects of a task which are currently too hard for the child (scaffolds the interaction). The scaffold is gradually reduced as the child is ready to take on more aspects of the task, until they can solve the task alone (without scaffolding). An example is a child constructing a pyramid with the help of an adult (Wood et al., 1976). At first, the child is not even focusing on the task, and the adult tries to get its attention to the task by connecting blocks and building the pyramid in front of them. Once the child is able to focus on the task, the adult starts passing the blocks to the child to connect. In the next phase, the child is grabbing blocks by itself, and the adult is helping through verbal suggestions. Then, only verbal confirmations are needed to guide the child. Finally, the child can construct the pyramid by itself. In summary, the adult observes the child and gradually transfers parts of the task (removes the scaffold) to the child. Through this process, the caretaker enables the child to master a task they would not be able to master alone.

## 3 The SocialAI school

The SocialAI school is a tool for building interactive environments to study various questions regarding social competence, such as “What do concepts related to social abilities and motivations (outlined by developmental psychology) mean in the scope of AI?”, “How can we evaluate their presence in different AI agents?”, “What are their simplest forms and how can AI agents acquire them?”

To construct SocialAI, we rely on a set of key experiments and studies from developmental psychology, which were used to outline the most important abilities, motivations and developmental steps in humans. From the work of Tomasello, we focus on developments before and around the age of 9 months (we believe it is important to address those before more complex ones relating to development of 3-year-olds, see Section 2.1). We study the following developmental pathways: Social cognition (inferring other's perception and joint attention), Communication (referential communication through the pointing gesture and the beginning of conventionalized communication through simple language), and Cultural Learning (imitation and role reversal imitation). From the work of Bruner, we study the concepts of Formats and Scaffolding (see Section 2.2). Using The SocialAI school, we construct environments and conduct experiments regarding all of those concepts.

SocialAI, which is built on top of Minigrid (Chevalier-Boisvert et al., 2018), includes a *customizable parameterized* suite of *procedurally* generated environments. We implement this procedural generation with a tree-based structure (the parametric tree). This makes it simple to add and modify new environments, and control their sampling. All the current environments are single-agent and contain a scripted peer. The agent has to interact with the peer to reach an apple. This setup enables a controlled and minimal representation of social interactions. To facilitate future research, this tool was made to be very easy to modify and extend. The SocialAI school is completely open sourced, and we hope that it will be useful to study the questions regarding social intelligence in AI.

The remainder of this section is organized as follows. First, Section 3.1 describes technical details such as the observation and the action space. Then, Section 3.2 introduces the parameter tree and explains how it can be used to sample environments. Finally, Section 3.2 describes two environment types, which were used in case studies in Section 4. In the Supplementary material, we present one additional environment type.

### 3.1 Parameterized social environments

The SocialAI school is built on top of the MiniGrid codebase (Chevalier-Boisvert et al., 2018), which provides an efficient and easily extensible implementation of grid world environments. SocialAI environments are grid worlds consisting of one room. In all of our environments, the task of the agent is to eat the apple, at which point it is rewarded. The reward is diminished according to the number of steps it took the agent to complete the episode. The episode ends when the agent eats the apple, uses the *done* action, or after a timeout of 80 steps.

**Figure 3:**
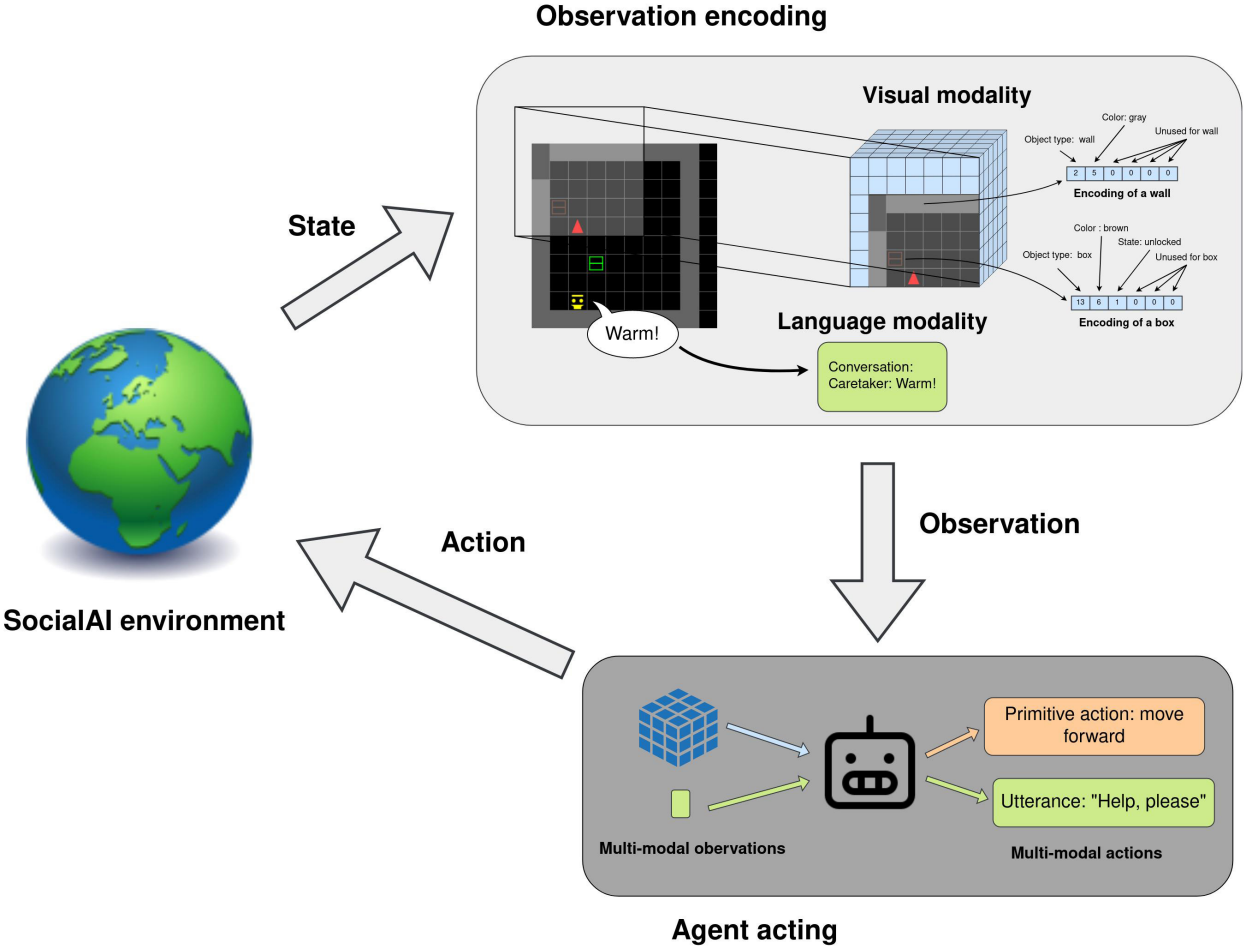
Workflow of an agent acting in the SocialAI school. The environment generates a state, which is represented as multimodal observations: a 7 × 7 × 6 tensor and the full dialogue history. The agent acts through a multi-modal action space consisting of primitive actions and utterances.

The agent's observation and action spaces are shown in Figure 3. This multimodal observation space consists of the full dialogue history, and a 7 × 7 × 8 tensor corresponding to the 7 × 7 grid in front of the agent. Each cell is encoded by six integers representing the object type, color, and some additional object-dependent information (e.g. is the door open, point direction, gaze direction, etc). A list of all possible objects in provided in the Supplementary material. The agent acts in the environment through a multimodal action space, which consists of 6 primitive actions (*no*, movement actions, *toggle*, and *done*) and a 4 × 16 templated language (the agent also has the option not to speak). All environments can also be instantiated as Textworlds (Côté et al., 2018). This procedure is explained in more detail in Section 4.5.

**Figure 4:**
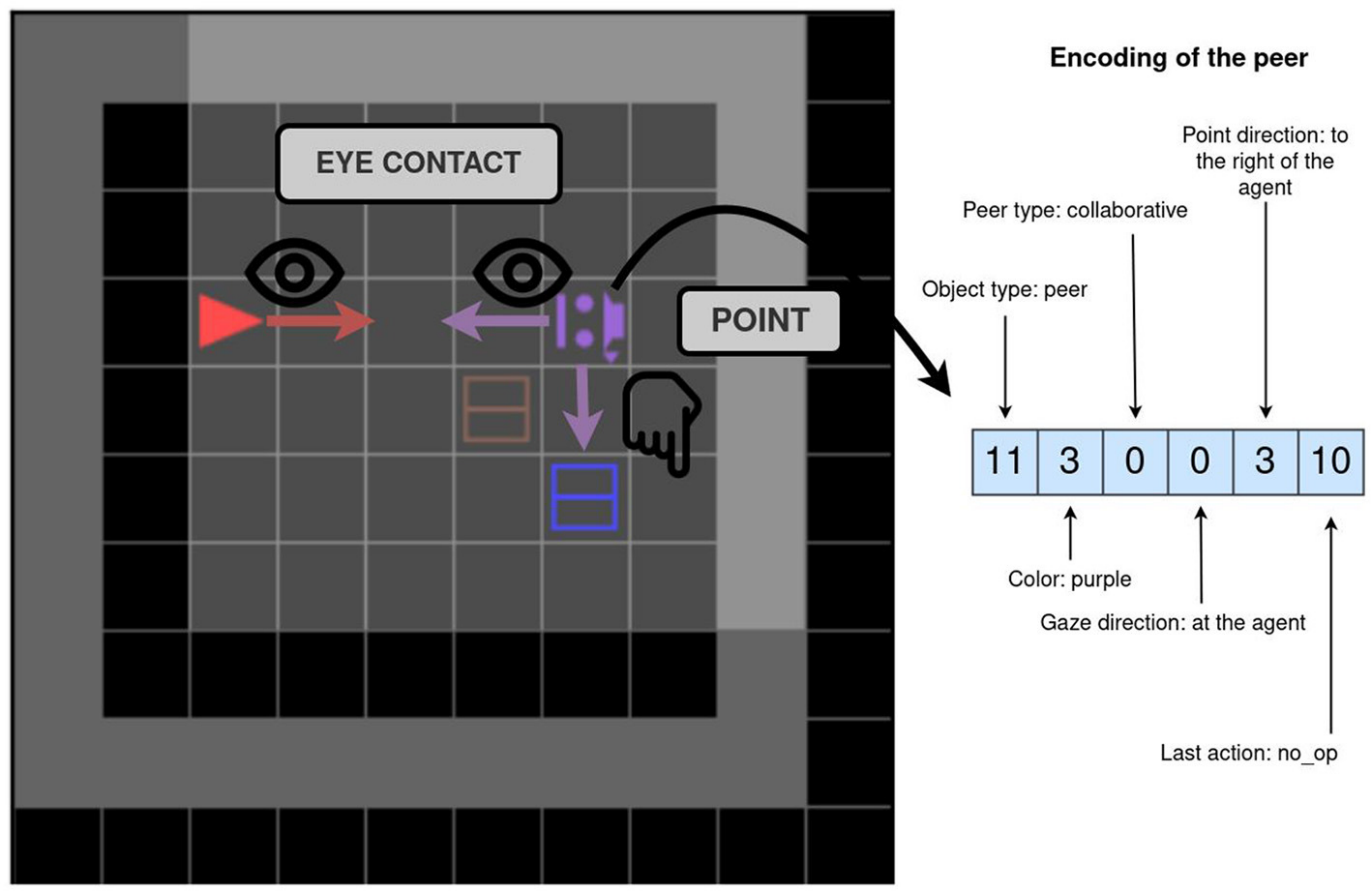
A depiction of a peer and its encoding. The agent and a peer are in eye contact, and the peer is pointing to the blue box. To the right is an encoding of the peer. The encoding contains information about the peer, e.g. the gaze and point direction.

All environments, unless otherwise stated, contain a scripted social peer, and the task can only be solved by interacting with this peer (for which socio-cognitive abilities are needed). A social peer observes the world in the same way as the agent does (as a grid in front of it), and it also observes the agent's utterances. Their action space consists of primitive actions for movement, pointing, and the *toggle* action. The peer can also communicate with words and sentences. As the peer is scripted, there are no constraints on the language it can utter (it is not constrained to a templated language). The language it uses depends on the environment, which defines with sentence the peer will utter at which point. The peer is represented in the agent's observation by seven integers depicting their: object type, position, color, type (cooperative or competitive), gaze direction, point direction, and the last executed primitive action. The peer's gaze and point directions are represented relative to the agent (e.g. 1—to the left of the agent). The pointing direction can also be set to 0, which signifies that the peer is not pointing. Figure 4 shows an example of an environment with the corresponding encoding of the peer. The agent (red) and the scripted peer (purple) are making eye contact—the peer and the agent are in the same row or column and their gazes meet frontally. In this example, the scripted peer is also pointing to the blue box.

The SocialAI environments are parameterized, and those parameters define the social dimensions of the task. In other words, parameters define which socio-cognitive abilities are needed to solve the task. For example, depending on the Environment type parameter, the peer can give information, collaborate with the agent, or be adversarial. In the case of the peer giving information, additional parameters define what is the form of this information (linguistic or pointing).

### 3.2 Parameter tree

**Figure 5:**
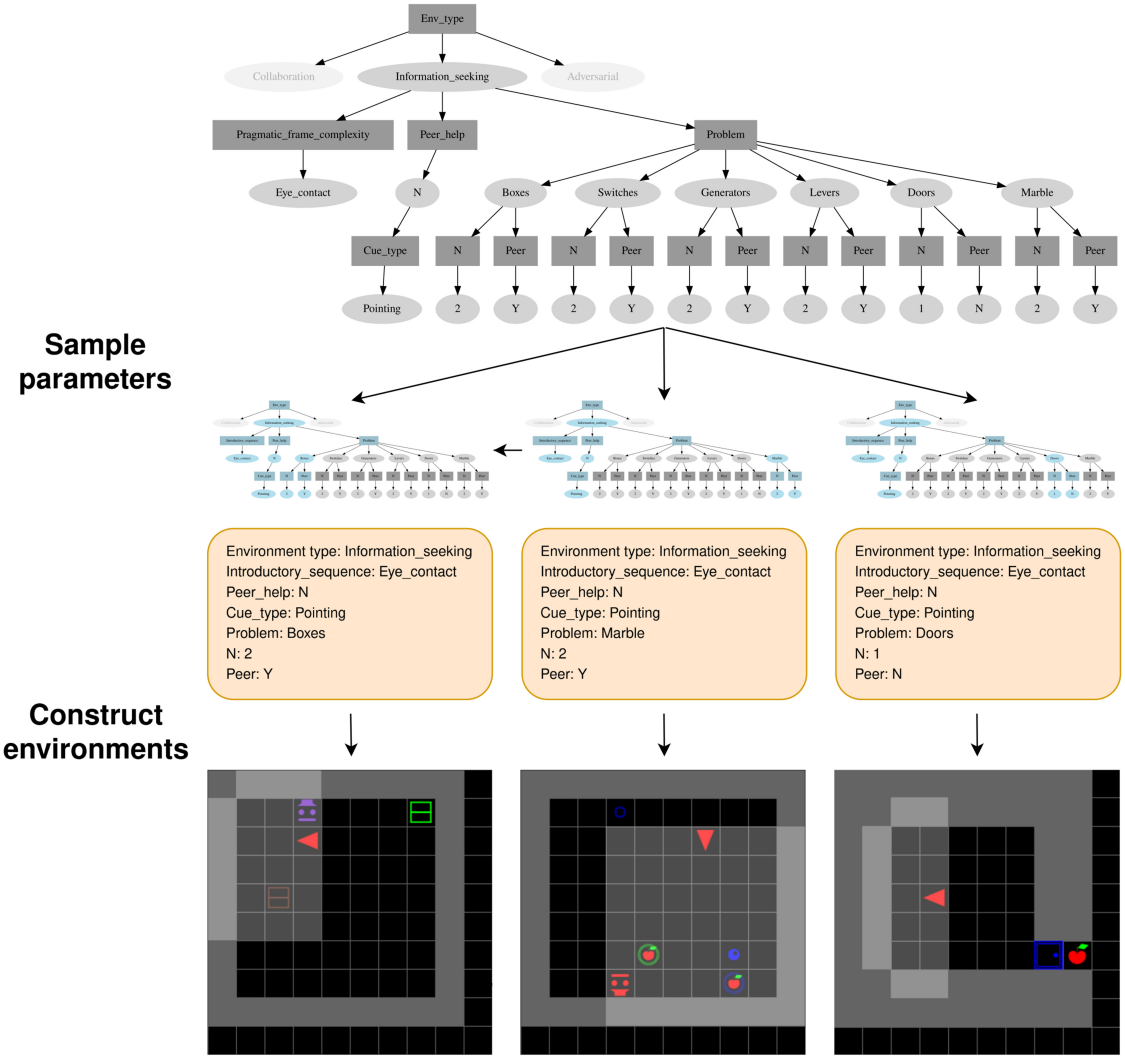
An example of procedural environment generation using tree-based parametric sampling. Parameter nodes (rectangles) require that one of its children (a value node) is selected. Value nodes (ovals) require that sampling progresses through all of its children (parameter nodes). Three examples of parameter sampling (and the three corresponding environments) are shown.

SocialAI enables the creation of many parameterized environments, and those parameters are implemented as nodes in a parameter tree. A parameter tree is a structure through which the experimenter can easily define which parameters (and their values) can be sampled. An example of such a tree can be seen in Figure 5. The experimenter defines a parameter tree at the beginning of the experiment. Each episode begins with the sampling of a set of parameters from this tree. Then, an environment is created and the agent placed inside.

The parameter tree is used to sample parameter sets, an example of such sampling is shown in Figure 5. There are two kinds of nodes: parameter nodes (rectangles) and value nodes (ovals). Parameter nodes correspond to parameters, and value nodes corresponds to possible values for those parameters. Sampling proceeds in a top-down fashion, starting from the root node. In all our experiments, Env_type parameter node is the root. Sampling from a parameter node selects one of its children (a value node), i.e. sets a value for this parameter. This can be done by uniform sampling over the node's children, or by prioritized sampling with a curriculum. Once a value node has been chosen, the sampling continues through all of its children (parameter nodes). In other words, setting a value for one parameter, defines which other parameters (the value node's children) need to be set. In our codebase, it is simple to create such trees, and add additional parameters and environments. In the following sections, we explain the most relevant parameters. The Supplementary material contains additional examples of parametric trees.

### 3.3 Environment types

The most important parameter is the environment type—Env_type (the root node). We implemented three different environment types: InformationSeeking, Collaboration, and AdversarialPeer. A parameter tree doesn't have to contain all of them, rather this depends on the type of experiment one wants to conduct (most often only one will be present). For example, Figure 5 shows the tree with only the InformationSeeking environment type. This tree was used to study understanding of the pointing gesture in Section 4.2. In the rest of this section, we describe the InformationSeeking and the Collaboration environment types. The AdversarialPeer type is described in the Supplementary material.

#### 3.3.1 Information seeking type environments

**Figure 6:**
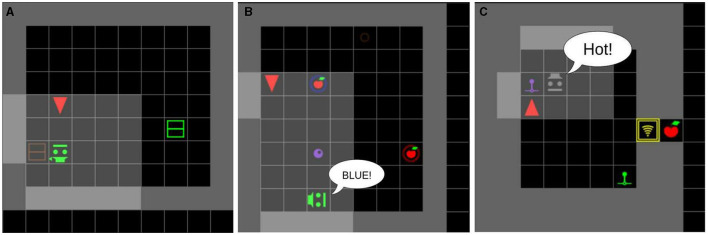
Examples of InformationSeeking type environments, in which agents learn to find hidden apples using textual or non-verbal communication with social peers. **(A)** A scripted peer pointing to a box. The agent needs to open the red box. **(B)** A scripted peer uttering the color of the correct generator. The agent needs to push the marble onto the blue generator. **(C)** A scripted peer hinting the distance to the correct lever (“Hot” means very close). The agent needs to pull the purple lever to open the door.

We used this environment type in case studies regarding communication, joint attention, and imitation learning. Figure 6 shows examples of InformationSeeking type environments.

The general principle of this environment type is as follows. The agent is rewarded upon eating the apple, which is hidden. The apple can be accessed by interacting with an object. The Problem parameter defines which objects will in the environment. There are six different problems: boxes, switches, marble, generators, doors, or levers. Different objects make the apple accessible in different ways. For example, opening the box will make the apple appear at the location of the box, while pulling the lever will open the door in front of the apple. A distractor can also be present (if *N* is set to 2). A distractor is an object of the same type as the correct object, but if it is used, both objects are blocked and the apple cannot be obtained in this episode.

To find out which object is the correct one, the agent must interact with the scripted peer. This interaction starts with the agent introducing itself. The way in which the agent should introduce itself is defined by the Introductory sequence parameter. We define the following four values: No, Eye_contact, Ask, Ask-Eye_contact. For the value No, no introduction is needed and the peer will give information at the beginning of the episode. In most of our experiments, we will use the value Eye_contact. For this value, the scripted peer will turn to look at the agent and wait for the agent to look at it. The agent must direct its gaze directly toward the scripted peer. An example of an established eye contact can be seen in Figure 4. For the value Ask, the agent needs to utter “Help, please” (a full grammar of the language is given in the Supplementary material). Finally, the Ask-Eye_contact value is a combination of the previous two (the agent utters “Help, please” during eye contact).

Once the agent introduces itself, the Help parameter defines the peer's behavior. If it is set to Y the peer with obtain the apple, and leave it for the agent to eat. Alternatively, it will give cues to the agent about which object to use. The nature of this cue is defined by the Cue type parameter. We define four different values: Pointing, Language Color, Language Feedback, and Imitation. For the Pointing type, the peer will point to the correct object. It will move to a location from which it can unambiguously point (e.g. the same row) and point to the object. For the Language Color type, the peer will say the color of the correct object. For the Language Feedback type, the peer will hint how close the agent is to the correct object. Every step, the peer will say “Cold,” “Medium,” “Warm,” or “Hot,” depending on how close the agent is to the correct object (e.g. “Cold” means that the agent is far from the object, and “Hot” that it is right next to it). For the Imitation type, the peer will demonstrate the use of the correct object. The peer will use the correct object, obtain the apple, eat it, and reset the environment to its initial state.

For the purpose of analyzing the agent's behavior more thoroughly, Information seeking environments can also be created without the distracting object, i.e. in their asocial versions. This can be achieved by setting parameter Peer to *N* and parameter *N* to 1. The asocial version of an information seeking environment contains no distractor, and no peer, i.e. the agent just needs to use the only object in the environment.

#### 3.3.2 Collaboration type environments

We used this environment type to study the agent's role-reversal ability. It consists of collaborative activities with two clearly defined roles. Environments are separated into two halves by a fence over which the agent can see, but which it cannot cross (each half corresponds to one role). If both roles are fulfilled correctly, two apples will become accessible (one on each side of the fence).

**Figure 7:**
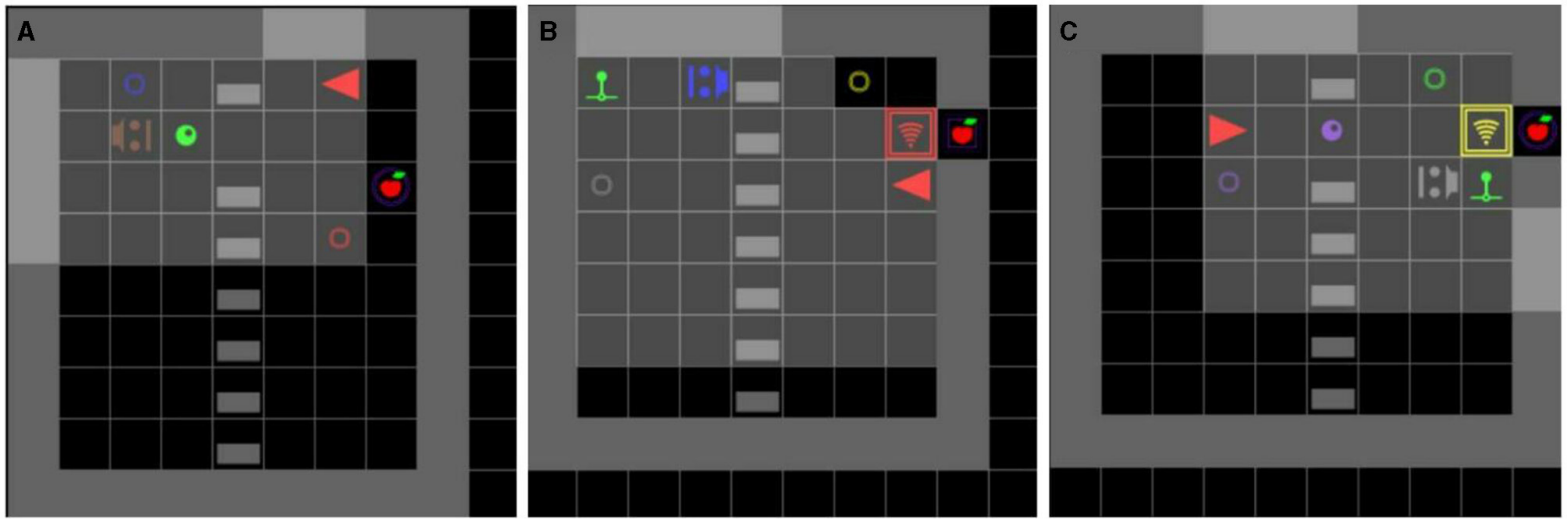
Examples of Collaboration type environments, in which agents must learn cooperative strategies with a (scripted) peer to achieve two-player puzzles. **(A)** The MarblePass problem with the agent in role B. The peer pushes the marble to the right and then the agent pushes it further to the purple *marble generator*. This makes two apples appear on the blue and red platforms. **(B)** The LeverDoor problem with the agent in role B. The peer opens the red door by pulling on the green lever. This enables the agent to go through the door and activate the purple generator This makes two apples appear on the gray and yellow platforms. **(C)** The MarblePush problem with the agent in role A. The peer opens the yellow door using the green lever. Then the agent pushes the marble through the door to the purple *marble generator*. This makes two apples appear on the purple and green platforms.

The most important parameters are Role and Problem. The Role parameter defines in which role to put the agent. The Problem parameter defines the collaborative activity, of which we implemented seven: DoorLever, MarblePush, MarblePass, Boxes, Switches, Generators, Marble. In DoorLever one participant opens the door by pulling the lever and the other passes through them, and activates the generator (generating two apples). In MarblePush one participant opens the door by pulling the lever, and the other pushes a marble through them. This marble activates the *marble generator* upon touching it. In MarblePass one participant pushed the marble to the right side of the room, and then the other redirects it toward the *marble generator*. In the other four problems, one participant is presented with two boxes of different colors, and the other participant is presented with two objects of those same colors and of the type defined by the problem parameter (e.g. two generators). First, the participant that was presented with boxes opens one box (an apple will be in both). After this, to obtain its apple, the other participant must use the object of the same color as the opened box. Figure 7 shows examples of Collaboration type environments.

Like the information seeking environments, collaboration environments can also be instantiated in their asocial versions. This can be achieved by setting the Version parameter to Asocial. The peer is not present in the environment, and the environment is initialized so that the task can be solved alone. For example, in MarblePass the marble is already on the right side of the room, so the agent just has to push it toward the *marble generator*.

## 4 Experiments

In this section, we demonstrate the diversity of experiments that can be conducted with the SocialAI school. To facilitate future research, the SocialAI school is easy to modify and extend, and is completely open sourced. We hope that it will be useful to study various questions regarding social intelligence in AI. Here, we present a series of such case-studies inspired by theories and studies discussed in Section 2.

The remainder of this section is organized as follows. In Section 4.1 we describe the agents used in case studies with reinforcement learning. In Section 4.2 we evaluate the generalization of socially recursive inferences by RL agents to new contexts—pointing in a new context. In Section 4.3 we show how an experiment from cognitive science can be recreated in the context of AI—we study the transfer of knowledge from one role to another, i.e. role reversal. In Section 4.4 we study how an RL agent can be made to learn a complex task by changing the environment (scaffolding) rather than the agent. Finally, in Section 4.5 we show how SocialAI environments can be easily transformed to pure Textworlds to study large language models as interactive agents. Five additional case studies are briefly outlined in Section 4.6 and presented in detail in the Supplementary material. These regard linguistic communication, joint attention, meta imitation learning, inferring the other's field of view, and formats (pragmatic frames). The Supplementary material also presents a pilot study, which was used to outline the most promising agent for all case-studies.

### 4.1 Baselines

In all of our case studies, except the study with LLMs (Section 4.5), we use a PPO (Schulman et al., 2017) reinforcement learning agent as depicted in Figure 3. The multimodal observation space consists of a 7 × 7 × 6 tensor (vision) and the full dialogue history (language). The multimodal action space consists of 6 primitive actions (no_op, turn left, turn right, go forward, toggle, and done), and a 4 × 16 templated language. The architecture of the agent is taken from Hui et al. (2020) and adapted for the multimodal action space with an additional output head (see Supplementary material for details). This additional head consists of three outputs: a binary output indicating if the agent will speak, and outputs for the template and the word to use.

In a set of pilot experiments (see Supplementary material) we proposed two count-based exploration bonuses, which we compared to other exploration bonuses including RND (Burda et al., 2018) and RIDE (Raileanu and Rocktäschel, 2020). Visual count-based exploration bonus (“PPO-CB”) performed best on the tasks in which language is not used, and its linguistic variant “PPO-CBL” performed best in environments with the peer giving linguistic cues. We therefore used those two agents in our case studies. Both of those two exploration bonuses are episodic. They estimate the diversity of observations in an episode and give reward proportional to that diversity. The linguistic exploration bonus uses the number of different words, and the vision-based exploration bonus the number of different encodings observed. In case studies in Sections 4.2, 4.3 we use the “PPO-CB” exploration bonus. The case study in Section 4.4 requires as raw PPO agent, and the one in Section 4.5 uses LLMs as agents.

### 4.2 Understanding the pointing gesture

This experiment is motivated by a study of children's ability to understand pointing gestures (Behne et al., 2005), discussed in Section 2.1.2. We study if an RL agent (with a visual count-based exploration bonus) can infer the meaning of a pointing gesture, and generalize this ability to new situations (infer the new meaning of a pointing gesture in a new context). This kind of generalization is relevant because the power of inferring pointing gestures is based on being able to infer its meaning to *new* referents based on *new* social contexts. We show how the SocialAI school can be used to evaluate the presence of generalizable social skills in artificial agents.

The environment consists of two objects (ex. boxes) and the peer that points to the correct object. The agent then has to interact with that object (ex. open the box) to get access to an apple. The agent is trained on five problems each with different objects (Boxes, Switches, Levers, Marble, Generators), and on the *asocial* version of the Doors problem (only one door and no peer). Training on the asocial version enables the agent to learn how to use a door, which is a prerequisite for generalization of the pointing gesture to an environment with two doors. The agent is evaluated on the Doors problem in the social setting (two doors and a peer pointing to the correct one). The agent needs to combine the knowledge of how to use a door (learned on the asocial version of that problem), with inferring the meaning of the pointing gesture (learned on the other five problems), and generalize that to a new scenario where the peer points to a door. To succeed, it needs to do pragmatically infer the intended meaning of the point (a socially recursive inference).

**Figure 8:**
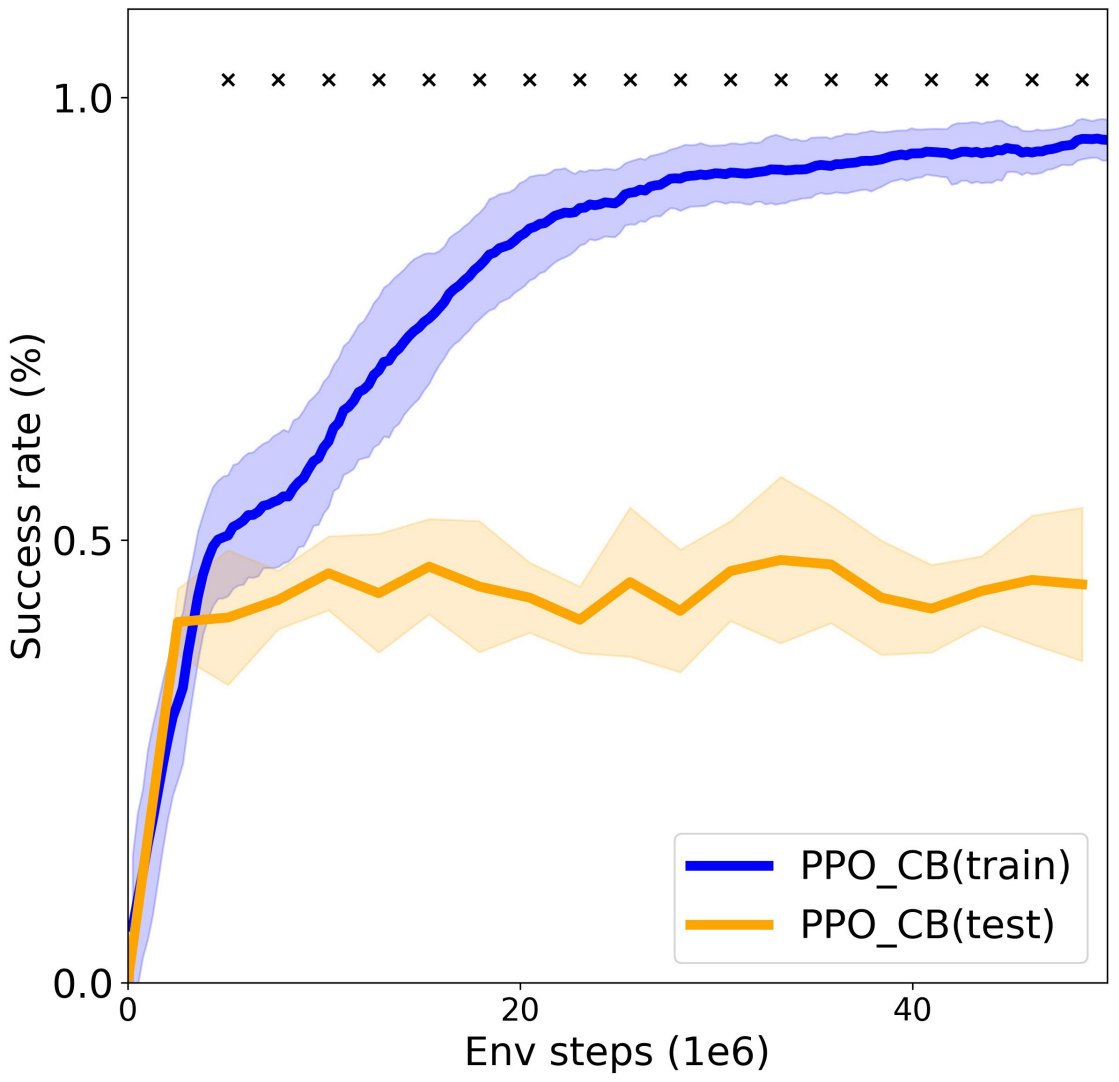
The Pointing experiments. Is an RL agent is able to infer the meaning of a pointing gesture in a new context? The figure compares the success rate (mean ± SD over eight seeds) on the training environments with the evaluation on the testing environment. The cross marks depict statistical significance (*p* = 0.05). The agent achieves high performance on the training environments, but it is not able to infer the meaning of a pointing gesture in a new context.

Figure 8 shows the success rate of the agent on the training environments [“PPO_CB(train)”] and on the evaluation environment [PPO_CB(test)]. We can see that while the agent easily solves the training environments (with the success rate of 95.2%), it fails to generalize to the testing environments (it reaches the success rate of 45.2%, which corresponds to randomly guessing the correct object). These results demonstrate that, while the agent can learn to infer the meaning of a pointing gesture in a familiar context, it cannot generalize to new social contexts. These results motivate future research on how an agent can be endowed with abilities for such combinatorial generalization. Given the recent advances in modeling social interactions with LLMs (Park et al., 2023), the LLM-based agents constitute a potential solution.

The Supplementary material presents similar two experiments in which the peer provides linguistic cues for the color and for the proximity of the correct object (instead of pointing). Similarly, we observe that, while the PPO agents master the training environments, they fail to generalize to a new context.

### 4.3 Role reversal imitation

In this experiment, we study the role-reversal capabilities of an RL agent: to what extent can it learn about the partner's role from playing its own. In doing so, we also show how SocialAI can be used to adapt a cognitive science methodology for AI to recreate the experiment. In Fletcher et al. (2012) apes and children were trained on one role (role B), and then tested on how long it took them to master the opposite role (role A). Results showed that children, but not apes, master role A faster than the control group (not pretrained). These results imply that children learn about the opposite role just from playing their own, i.e. they see the interaction from a bird's eye perspective. We study the following two questions: (1) How much do RL agents learn about the partner's role during a collaborative activity? (2) Does increasing the training diversity (training on more tasks in both roles) enable the agent to learn more about the partner's role?

We conduct this study on the MarblePass task. This task consists of two roles: one participant pushes the marble to the right side of the environment (role A), from where the other can redirect it toward the generator (role B). We study how much an agent learns about the testing role (role A), from the training role (role B). Following Fletcher et al. (2012) we measure the sample efficiency of fine-tuning agents to the test role. Unlike in Fletcher et al. (2012) it is not sufficient to compare an agent pretrained on the training role with an unpretrained agent. Even if the agent pretrained on the training role learns nothing about the testing role, it would still learn about environment dynamics and one would expect it to learn faster than the unpretrained agent. For this reason, we compare with an agent pretrained on the asocial version of the training role. In this version, the agent obtains reward in the same way as in the social version, but no peer is needed—the agent and the marble are placed on the right side of the environment and the agent has to push the marble toward the generator. Therefore, this agent learns all about the relevant environment dynamics, but not about the specific collaborative activity (this represents the control group in Fletcher et al., 2012).

We conduct two experiments: *single* and *group*. In *single* experiments, the agents are trained only on one problem: role B and the asocial version of the MarblePass problem. In *group* experiments, both agents are also trained on both roles of six additional collaborative problems (a total of 13 environments). In other words, we compare the agents pretrained in the four following ways: (1) experimental (*single*): pretrained only on role B of the MarblePass problem, (2) control (*single*): pretrained only on the asocial version of the MarblePass problem, (3) experimental (*group*): pretrained on role B of the MarblePass problem, and on both roles of all other problems, (4) control (*group*): pretrained on the asocial version of the MarblePass problem, and on both roles of all other problems.

#### 4.3.1 How much do RL agents learn about the partner's role during a collaborative activity?

**Figure 9:**
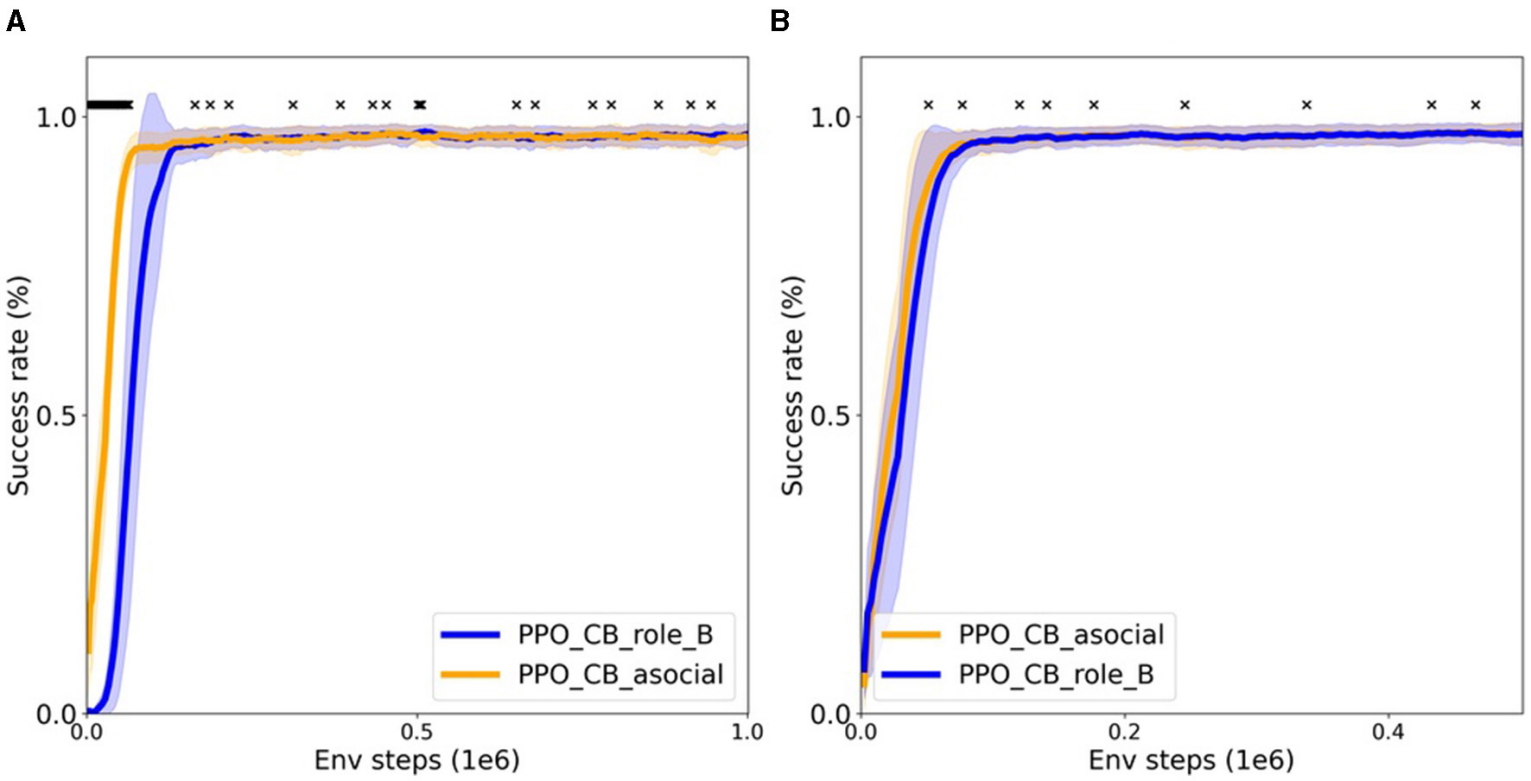
Role reversal imitation experiments. To what extent is an RL agent able to transfer knowledge from one role of a collaborative activity to another? The success rate of fine-tuning to role A (mean ± SD over nine seeds) is shown [x denotes statistical significance (*p* = 0.05)] Agents pretrained on role B do not master role A faster than asocially pretrained agents, implying that the RL agents do exhibit role reversal capabilities. **(A)** *Single* experiment: learning role A given pretraining on role B (one environment). **(B)** *Group* experiment: learning role A given pretraining on role B and six other two-roles tasks (13 environments).

Figure 9A shows the success rate of fine-tuning to role A of the MarblePass task. It compares the experimental and the control conditions of the *single* experiments. It is interesting to note that the agent pretrained on the asocial version (“asocial”) masters role A of the task slightly faster than the agent pretrained on role B of the task (“role_B”). This implies that, not only, the agent does not learn anything useful about the peer's role, but pretraining on role B actually makes it harder for the agent to learn about role A. We believe that this is because, during training in role B, the agent learns to first wait for the peer, while in the asocial version it pushes the marble right away. As, in role A, the agent pushes the marble right away too, we believe this makes it slightly easier for the asocially pretrained agent to adapt to the new role. In other words, from an egocentric view the asocial version is closer (than role B) to role A. This shows that the RL agent, rather than understanding the interaction from a bird's-eye perspective, finds the simplest way to solve the task.

#### 4.3.2 Does training on additional problems enable the agent to learn more about the partner's role?

Figure 9B shows the success rate of fine-tuning to role A of the MarblePass task. It compares the experimental and the control conditions of the *group* experiments. Here we can see that there is no significant difference in sample efficiency. We can make two observations from this. First, as the socially pretrained agent was less sample efficient in the *single* experiments, we can conclude that pretraining on many tasks reduces overfitting on role B. And second, as this agent is not more sample efficient than the asocially pretrained baseline, we can conclude that this agent does not learn anything useful about the peer's role as well.

These results imply an interesting avenue of research into how agent's attention can be directed to the partner's role and the birds-eye-view of the activity.

### 4.4 Scaffolding

In this section, we study the concept of scaffolding (see Section 2.2 for details). We show how modifying the environment can make it easier for the agent to learn a complex task, i.e. we explore if a scaffolded environment can help an agent learn more complex interaction sequences (formats). This can be seen in contrast to the standard approach, where the environment is kept fixed and the agent improved (e.g. with an exploration bonus). For this reason, here we use a PPO agent without an exploration bonus. From the AI perspective, scaffolding can be seen as analogous to curriculum learning (Bengio et al., 2009). In curriculum learning, the task is made gradually more complex, enabling the learner to gradually acquire it part by part. Scaffolding refers to the caretaker taking a large part of the task on itself, and then gradually, as the learner becomes more proficient, transferring parts of the task to the learner until the learner can do the whole task by themselves. We show how the SocialAI school can be used to study curricula fostering social skill acquisition.

The environment is similar to the one in Section 4.2 with small changes. First, we evaluate on all six problems (instead of one) in the social version. Second, instead of pointing, the peer gives linguistic cues for how close the agent is to the target object (e.g. “Hot” for very close). And third, these cues are given after a more complex introductory sequence (established eye contact and the utterance of “Help, please”). The agent is trained in two phases. In the first phase, the agent is trained on environments of different complexity. After reaching a set success rate the training goes to the second phase. In the second phase, the agent is trained only on the six testing environments. We compare two types of scaffolding: “scaf_4” and “scaf_8.” The scaffolding type defines the environments in the first phase. The “scaf_4” agent is trained on four different introductory sequences (requiring or not requiring eye contact and the utterance)—a total of 18 environments (six problems, four sequences). The “scaf_8” agent is in addition trained on environments where the peer can help in two different ways: linguistically hinting to the object or interacting with it and leaving the apple for the agent to eat—a total of 36 environments (six problems, four sequences, two helping options). The easiest environments on which the “scaf_8” agent is trained do not require an introduction and the peer leaves the apple for the agent, i.e. the agent just goes to the apple and eats it. The hardest ones require the introduction with both the utterance and the eye contact, and include the peer linguistically hinting to the object (those hardest environments constitute the testing set).

**Figure 10:**
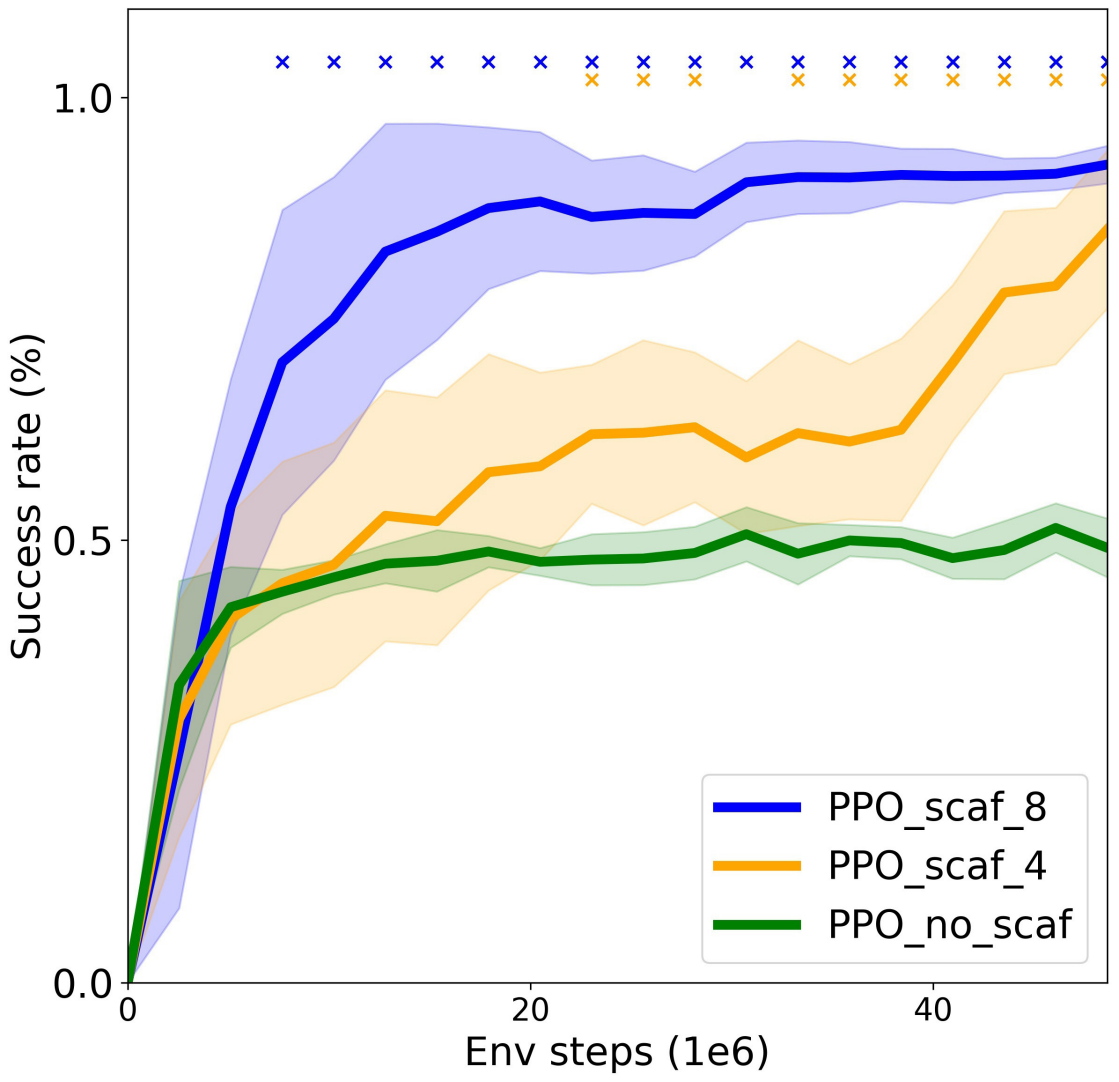
Scaffolding experiment. Comparing agents trained on multiple environments of varying difficulty to an agent trained an unscaffolded environment. Success rates on the testing environments (mean ± SD over eight seeds) are shown [x denotes statistical significance (*p* = 0.05) with respect to the “no_scaf” baseline]. Only the scaffolded agents solve the environments (scaffolding with eight difficulty levels is the best).

Figure 10 compares the success rate of the agents trained with the two scaffolding types (“scaf_4” and “scaf_8”) to that of an agent trained only on the six testing environments (“no_scaf”). We can see that only the scaffolded agents solve the testing environments, and that the agent with a more detailed scaffolding (“scaf_8”) solves the environment faster. These results show that scaffolding enables the agents to learn more complex formats, and that a more thorough scaffolding further improves the efficiency. In future work, more advanced scaffolding could be explored [e.g. based on learning progress (Oudeyer and Kaplan, 2007) or other surrogate objectives (Portelas et al., 2020)].

### 4.5 Large language models as interactive agents

Large language models (LLMs) are starting to be used in various tasks (Brown et al., 2020; Devlin et al., 2018; Zhang et al., 2022; Ouyang et al., 2022), including to control interactive agents (Yao et al., 2022; Carta et al., 2023). In this section, we show how the SocialAI school enables the parsing of visual grid observations to text in order to study LLMs as interactive agents This process can be easily modified, which simplifies prompt engineering (Liu et al., 2021) and similar experimentation.

**Figure 11:**
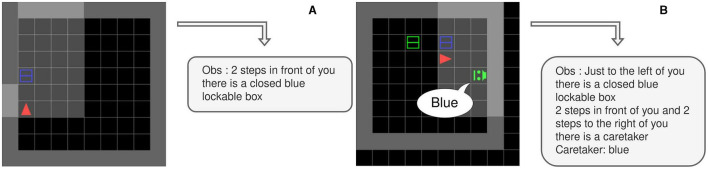
Environments used in the experiments with LLMs with observations are parsed into text. **(A)** The AsocialBox environment **(B)** The ColorBoxes environment.

We use two environments: AsocialBox and ColorBoxes (see Figure 11). The ColorBoxes enviroment is in addition used to test generalization. In the AsocialBox environment, there is a box, which the agent has to open to get the apple. In the ColorBoxes environment, there are two boxes and a peer. At the beginning of the episode, the peer utters the color of the box with the apple. When testing for generalization, we create in-context examples in environments with other objects (e.g. doors, levers) and in the asocial version of the Boxes problem (analogous to the training environments in Section 4.2). To generalize, an agent must infer the meaning of the peer's utterance in a new context (to select the correct box) and combine this with the knowledge of how to open a box (from the asocial version).

**Figure 12:**
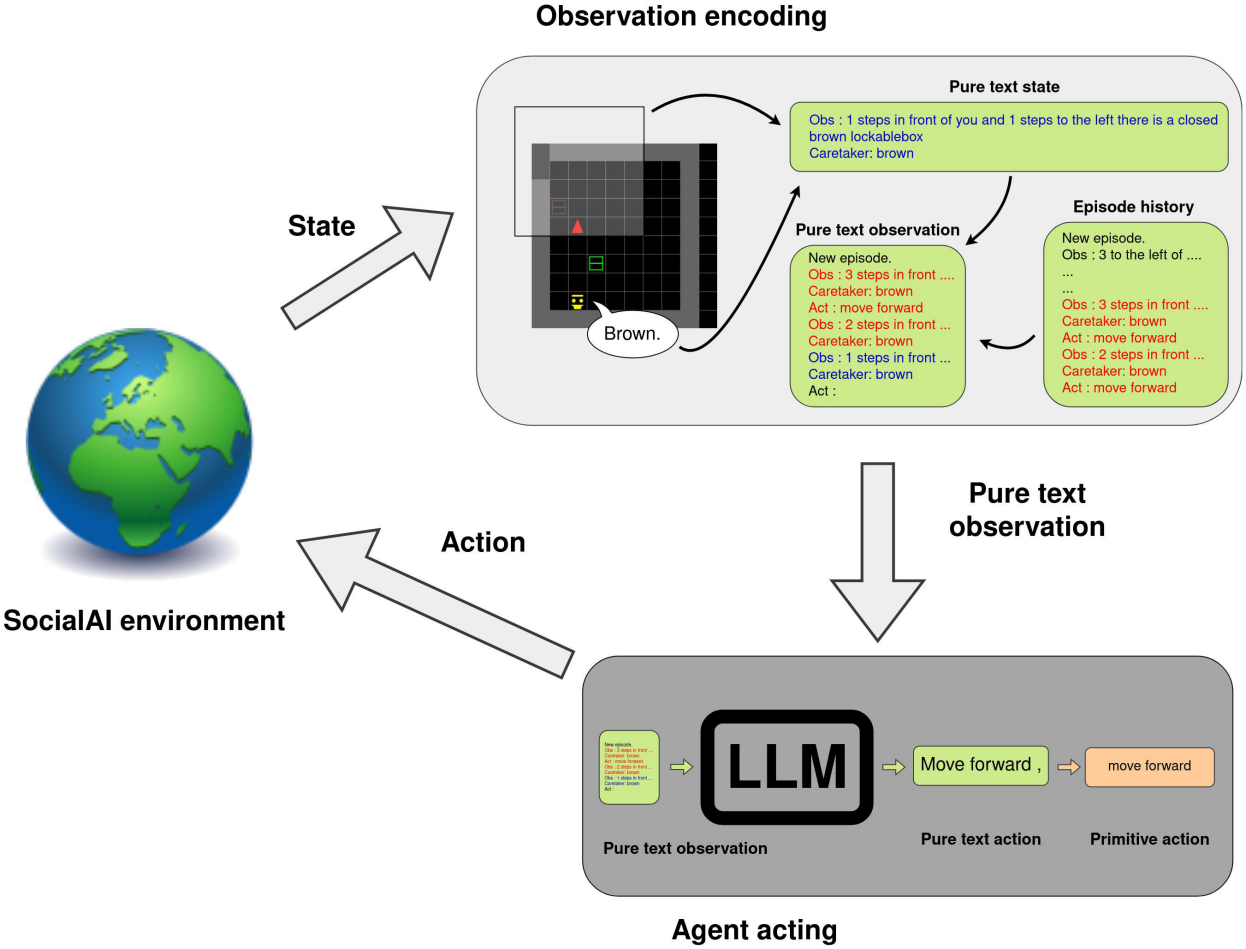
An example of how a language model can be used as an interactive agent. An observation is parsed into text and combined with previous two observations and actions. This is appended to the in-context examples, and is used as prompt for the LLM. The agent generates text that is matched (as case-insensitive substring) with the list of possible actions.

A language model acts by generating text given some textual prompt. In our experiments, the prompt contains the following: the in context examples, the last three steps (observations and actions) of the current episode, and the action query (“Act:”). The observations are parsed to text as shown in Figure 12. We manually create expert trajectories to be used as in-context examples—six episodes for the AsocialBox environment, and five for ColorBoxes (the full in-context examples are given in the Supplementary material. The model then generates the textual continuation of this prompt.^1^ If one of the available actions (“turn left,” “turn right,” “move forward,” “toggle”) is a substring of the generated text, the action is executed and the environment generates the next observation. However, if no action was matched to the generated text, the “no_op” action is executed (the agent does not act this step). The executed action and the new observation are then added to the prompt.

We compare six different large language models: the open-source multilingual bloom-560m (Scao et al., 2022) (560M), and five models from the GPT (Brown et al., 2020) family “text-ada-001” (estimated to be 350M^2^), “text-davinci-003” (175B parameters), “gpt-3.5-turbo-instruct-0913,” “gpt-3.5-turbo-0613,” and “gpt-4-0613.” We also compare with a random baseline, which samples a random action each step. We evaluate these models on a fixed test set of 10 environments for AsocialBox and 20 environments for ColorBoxes, with a time limit of 15 steps.

Table 1 shows that, on the AsocialBox environment, the best GPT models (gpt-4 and davinci-003) achieve a high performance (100% success rate), despite only observing six expert trajectories. On ColorBoxes, GPT-4 is the only model to achieve high performance (75%). This model escapes the local optimum of 50% (randomly choosing a box to open), these results imply that the model uses the given social cue (the peer's utterance of the color). As GPT-4 was the only model to do so, we test only this model on generalization. The model reaches a performance of 55%, which implies that the model doesn't generalize to a new social context—it randomly chooses a box to open.

**Table 1:** Comparison of LLM-based agents on two SocialAI environments parsed into pure text (see Figure 12).

	**gpt-4**	**gpt-3.5-turbo**	**gpt-3.5-turbo-instruct**	**ada-001**	**davinci-003**	**Bloom-560m**	**Random**
AsocialBox	**100%**	90%	90%	90%	**100%**	10%	0%
ColorBoxes	**75%**	5%	25%	0%	15%	5%	5%
ColorBoxes (generalization)	**55%**						

The best model (gpt-4) reached the success rates of 100% on AsocialBox, and 75% on ColorBoxes. The score of 75% suggests that the model is levering the peer to choose the correct box. When tested for generalization this model reached 55% success rate implying it is not able to generalize to a novel object. These expriments demontrate how LLM-based agents can be used in the SocialAI School. While more detailed analysis is needed to reach stronger conclusions, the performance is impressive given that the models observed only six (for AsocialBox) and five (for ColorBoxes) expert trajectories. We are confident that with more advanced LLM-based methods better performance can be achieved. The bold numbers indicate the best performing model for each task.

The motivation of this experiment was only to show how LLM-based agents can be studied in SocialAI. Therefore, more detailed experiments and analysis are needed to reach stronger conclusions. Even though the environments used in this case study are simpler than those RL case studies (only the Boxes problem, and no introductory sequence), we find it impressive that such performance is achieved from observing only a few expert trajectories: six for AsocialBox and five for ColorBoxes given that the model was not explicitly pretrained for such a task.

We are optimistic that in future work LLM-based agents could solve much more complex tasks with further prompt engineering and more advanced methods. Promising methods include planning (Huang et al., 2022), chain-of-thought reasoning (Wei et al., 2022; Zhang et al., 2023), fine-tuning (Ouyang et al., 2022; Carta et al., 2023), and many more. As the main motivation of this case study was to show that it is easy to study large language models with the SocialAI school, we leave those experiments for future work.

### 4.6 Additional experiments

We refer interested readers to the Supplementary material for details on additional case studies, which we briefly outline in this section. As mentioned in the pointing case study (Section 4.2), we performed analogous experiments to study whether the agent can leverage linguistic cues instead of the pointing gesture. We observe similar results: while the agents master the training environments, they fail to generalize to new context.

We study **joint attention** as defined by Tomasello (see Section 2). Environments feature a peer providing cues both inside and outside joint attention. Informative cues are only given inside joint attention (after completing the introductory sequence), while misleading random cues are given outside joint attention. In our experiments, the agent was unable to sufficiently discriminate between those cues to solve the task. We present a case-study on the acquisition of an (in-episode) **imitation learning** mechanism. From the AI perspective, this can be seen as social meta-learning: the agent acquires (through gradients) the imitation learning mechanism, which is used during the episode to learn an instrumental action on a new object. This study is motivated by an experiment from cognitive science in which children showed such imitation abilities (Carpenter et al., 1998). Experiments showed that RL agents are not able to acquire a learning mechanism which would enable them to learn how to use a completely new object at test time. We test the agent on its **ability to infer the peer's field of view**. The agent is rewarded for eating the apple without being observed by the peer. We show that the agent partially infers the peer's field of view, but is still not able to match the upper performance bound. Finally, we study the acquisition and use of **formats** as defined by Jerome Bruner (Section 2.2), i.e. protocols of social interactions. Agents were trained on tasks in which cues can be obtained from a peer after a more complex introductory sequence (Ask_Eye_Contact). The results show that, while an RL agent trained without the exploration bonus was unable to learn that introductory sequence, the agent with a linguistic count-based exploration bonus was. This results can be interpreted in tandem with the scaffolding case study (Section 4.4) in which an RL agent without an exploration bonus is able to learn the most complex introductory sequence, given training in a scaffolded environment. Therefore, the acquisition of complex formats can be achieved either by changing the learner or the environment. These additional case studies show further examples of interesting research questions that can be explored with the SocialAI school.

## 5 Conclusion and discussion

Following contemporary research in developmental psychology, this work presents and studies a wider set of socio-cognitive abilities than those usually studied in the field of AI. The motivation of this work is to introduce those concepts to AI and motivate related research. We present an introduction to Michael Tomasello's and Jerome Bruner's theories of socio-cognitive development. Following these theories, we outlined a set of key socio-cognitive abilities and concepts for AI: social cognition (inferring other's perception and joint attention), communication (referential and early conventionalized communication), cultural learning (imitation and role reversal imitation), scaffolding, and formats.

We introduce the SocialAI school—a tool simplifying the research of core socio-cognitive abilities. We show how the SocialAI school can be used to easily create environments studying various questions inspired by developmental psychology. With RL agents, we conduct experiments regarding the pointing gesture, scaffolding, and role reversal (by recreating an experiment from developmental psychology). We demonstrate that, by using SocialAI to parse environments into text, Large Language Models be easily studied as well. In the Supplementary material, we present additional studies concerning linguistic communication, joint attention, imitation learning, inferring others' field of view, and formats. Our experiments demonstrated the diversity of studies that can be conducted with the SocialAI school, highlighted the limitations of standard RL agents, and showed that while large language models learn with high sample efficiency, additional methods such as fine-tuning or chain-of-thought might be needed for generalization.

### 5.1 Limitations

In this work, we outline and discuss several concepts from developmental psychology (mostly regarding the development before and around 9 months of age), which we believe to be most relevant for AI at the moment. Due to the magnitude of this field of research, many relevant cognitive concepts are either discussed very briefly (e.g. conformity, social norms, instructed learning) or left unmentioned (e.g. morality, fairness, sense of self). Furthermore, while we argue that the work of Tomasello and Bruner provides an interesting framework to guide AI research in social skill acquisition, many other perspectives could have been considered as well, e.g. Erikson (1993), Gopnik and Meltzoff (1997), or Heyes (2019).

Similarly, as the present work merely represents a first step toward socially proficient artificial learners, many technical dimensions were simplified. In particular, we refrain from free form language dialogues and consider simple templated language. Likewise, we do not use human or trained peers, but scripted peers (which enables to isolate social abilities). Rather than implementing rich 3D visual worlds with continuous actions, we use grid-worlds with discrete primitive actions. We argue that such simplifying assumptions affords tractable studies while maintaining enough social complexity to model and isolate various social challenges. Assuming progress is made over these social scenarios, an interesting avenue for future work will be to extend the parametric generation toward environments with more complex sensorimotor challenges.

### 5.2 Future work

Given recent works showcasing the importance of Automatic Curriculum Learning in “asocial” DRL (Parker-Holder et al., 2022; Portelas et al., 2020), an interesting direction for future work would be to study whether this can also be observed in the SocialAI school. Our short case study on the importance of scaffolding (Section 4.4) suggests a positive impact, although we restricted our analysis to simple expert curricula. An interesting challenge is to design curriculum methods able to leverage the hierarchical structure of SocialAI's parametric tree, rather than the usual low-dimensional flat spaces of task-encoding parameters (predominant in the literature).

Large language models (LLMs) are spreading to countless branches of artificial intelligence. A promising avenue of future research is the application of language models to interactive agents (Carta et al., 2023). In this paper, we studied LLMs only on simple environments with a simple method—prompting the model with a few expert trajectories. While this approach showed impressive sample efficiency, it is limited due to the constraints on the prompt size. These experiments should be revisited with more powerful methods, such as fine-tuning or chain-of-thought prompting. Such methods could potentially make more complex social inferences, leading to better performance on many case studies in this paper, especially the ones related to generalization to new scenarios.

An important factor for the observed learning failures of our PPO agents in our case-studies might be linked to the simple forms of exploration bonuses that we used. Finding an exploration bonuses suited for social challenges is another interesting direction. We show that RIDE (Raileanu and Rocktäschel, 2020) and RND (Burda et al., 2018), two state-of-the-art exploration bonuses from classical DRL underperformed compared to our simple CountBased methods. An interesting avenue would be to study recent exploration bonus methods designed for social scenarios, e.g. Zhang et al. (2020).

## Data Availability

The code and additional resources (see Figure 2) of the SocialAI school can be found at the project website: https://sites.google.com/view/socialai-school/home.

## Footnotes

^1^We generate three tokens for GPT models, and three words for bloom.

^2^ https://blog.eleuther.ai/gpt3-model-sizes/
