# Supplementary material for: The SocialAI school: a framework leveraging developmental psychology toward artificial socio-cultural agents
Source: Front Neurorobot. 2024 Oct 9;18:1396359. doi: 10.3389/fnbot.2024.1396359 (PMC11496287; doi:10.3389/fnbot.2024.1396359)
Supplement: Supplementary file 1 [file Data_Sheet_1.PDF]

Supplementary Material

1 ARCHITECTURE OF THE RL AGENT

In this work, we use a PPO (Schulman et al., 2017) with an architecture initially designed for the BabyAI benchmark (Chevalier-Boisvert et al., 2019). The policy design was improved in a follow-up paper by Hui et al. (2020) (more precisely, we extend their *original_endpool_res* model). See figure S1 for a visualization of the complete architecture. First, symbolic pixel grid observations are fed into two convolutional layers (LeCun et al., 1989; Krizhevsky et al., 2012) (3x3 filter, stride and padding set to 1), while dialogue inputs are processed using a Gated Recurrent Unit layer (Chung et al., 2015). The resulting image and language embeddings are combined using two FiLM attention layers (Perez et al., 2017). Max pooling is performed on the resulting combined embedding before being fed into an LSTM (Hochreiter and Schmidhuber, 1997) with a $128D$ memory vector. The LSTM embedding is then used as input for the navigation action head, which is a two-layered fully-connected network with tanh activations and has an 6D output (i.e. 5 navigation actions and no_op action).

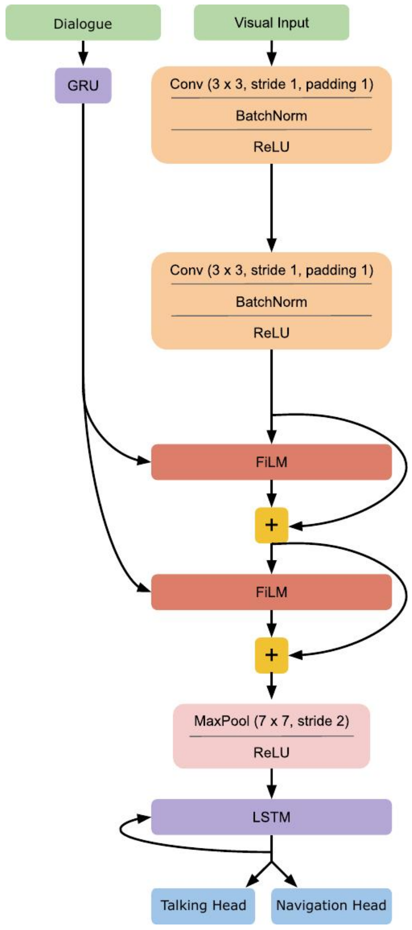

Figure S1. Our Multi-Headed PPO baseline DRL agent. Architecture visualization is a modified version of the one made by Hui et al. (2020). We perform two modifications: 1) Instead of fixed instruction inputs our model is fed with NPC's language outputs (if the agent is near an NPC), and 2) We add a language action head, as our agent can both navigate and talk.

In order for our agent to be able to both move and talk, we add to this architecture a talking action head, which is composed of three subheads. All of them consist of two fully-connected layers with tanh activations, and take the LSTM's embedding as input. The first one is used as a switch: it has a one-dimensional output to choose whether the agent talks (output > 0.5) or not (output < 0.5). If the agent talks, the two other subheads are used to sample the template and the word. Grammar of the templated language is depicted in table S1 and examples of multi-modal actions in table S2.

Note that the textual input given to the agent consists of the full dialogue history as we found it works better compared to giving only the current utterance.

2 EXPLORATION BONUSES

The used exploration bonuses are inspired by recent works in intrinsically motivated exploration (Pathak et al., 2017; Savinov et al., 2018; Tang et al., 2017). These intrinsic rewards estimate the novelty of the currently observed state and add the novelty based bonus to the extrinsic reward.

In this work we present two techniques for computing the count-based exploration bonus. Both of our count-based exploration bonuses are episodic - they estimate the diversity of states observed within an episode, and assume that beneficial episodes are those with more diverse observations.

Language-based exploration bonus (CBL) For some utterance s_{lang} observed at state s, we count how many times was this utterance observed during the episode. We compute the bonus for this step using the following equation:

$$r_{intr} = T * tanh\left(\frac{C}{(N(s_{lang}) + 1)^M}\right) \tag{S1}$$

, where M, C, and T are hyperparameters and $N(s_{lang})$ is the number of times the utterance s_{lang} was observed during this episode so far.

Vision-based intrinsic reward (CB) We reward the agent for observing diverse encodings. An encoding is the 6D representation of a cell. A visual observation consists of 47 (7x7) encodings representing cells in front of the agent. For some visual observation s_{viz} at step s, a set of encountered unique encodings is created (duplicates are removed) $U(s_{viz})$, and then the reward computed using the following equation:

$$r_{intr} = T * tanh\left(\sum_{e \in U(s_{viz})} \frac{C}{(N(e) + 1)^M}\right) \tag{S2}$$

, where M, C, and T are hyperparameters, $U(s)$ is a set of unique encodings visible in state s, and $N(e)$ is the number of times an encoding e was encountered in the current episode.

3 ADVERSARIAL ENVIRONMENT TYPE

In the main text, we discussed two environment types: INFORMATIONSEEKING and COLLABORATION. In this section, we explain an additional environment type - ADVERSARIAL type. This environment type is used to study the ability of the agent to infer the peer's field of view. An apple is present in the environment right away. However, the agent will get rewarded only if it eats it while not being observed by the peer (the peer is adversarial). Therefore, the agent needs to infer the right moment to eat the apple. The OCCLUSIONS parameter refers if the occlusions are present in the environment. Figure 7 shows this environment type without any obstacles (figure 7A) and with obstacles present (figure 7B). This is motivated by experiments in which apes were shown to eat only when not being observed by the alpha male (demonstrating their ability to infer if another's field of view (Hare et al., 2001)).

4 PILOT EXPERIMENTS

In this pilot experiment, we compare two exploration bonuses presented in section 2 to RIDE (Raileanu and Rocktäschel, 2020), RND (Burda et al., 2018), and to the agent without any exploration bonus. [1] We encode the peer using a mix of egocentric and allocentric vision - the peer's gaze and pointing direction

[1] We verify our implementation of RIDE and RND by recreating the results of those baselines on environments from Raileanu and Rocktäschel (2020).

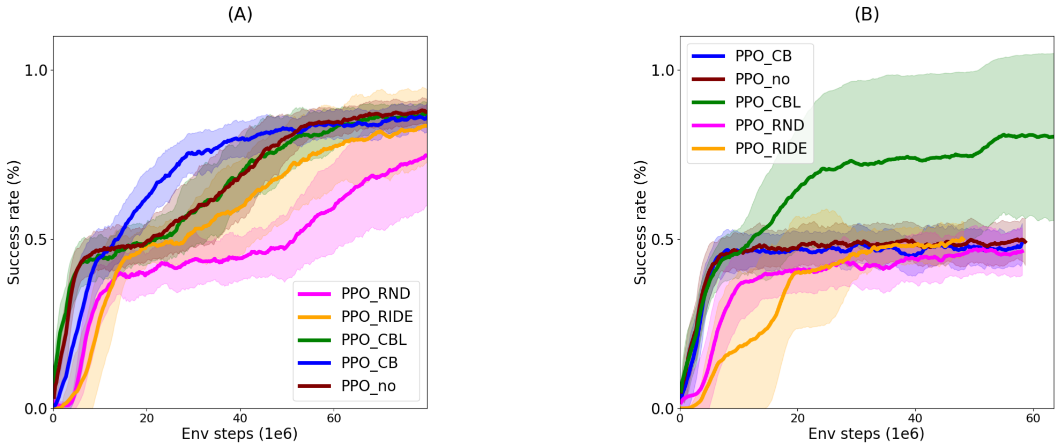

Figure 2. Pilot experiments showing that our count-based exploration bonuses outperform other baselines. On the environments with the pointing gesture, visual count-based ("CB") exploration bonus is the best performing condition. On the environments with utterances, linguistic count-based ("CBL") exploration bonus is the best performing condition. **(A)** Pilot experiments with the peer pointing to the correct object. **(B)** Pilot experiments with the peer uttering the color of the correct object.

were encoded in terms of absolute direction ("NSEW"). We changed this for other experiments as we found it more natural with regard to the question of socio-cognitive artificial intelligence. We believe that the best performing baselines would also perform best with purely egocentric encodings (the one we use in the rest of the paper). For that reason, and to avoid unnecessary energy spending, we do not compare with other baselines on the purely egocentric encoding.

Figure 2 compares PPO agents trained with different exploration bonuses discussed in section 2 on two different InformationSeeking type environments. The first environment involves the peer pointing to the correct object. Figure 2A shows that the best performing agent is the one levering the visual count-based exploration bonus (PPO_CB). The second environment involves the peer uttering the color of the correct object. Figure 2B shows that the best performing agent is the one levering the linguistic count-based exploration bonus (PPO_CBL). We conclude that PPO_CBL is the most suitable baseline for environments involving linguistic cues, and PPO_CB for the other environments.

5 DETAILS ON THE PARAMETER TREES USED IN THE MAIN TEXT

5.1 The Pointing experiment

The parameter trees used in this experiment are depicted in figure 14. We used the InformationSeeking environment type. The Introductory_sequence is set to Eye_Contact, and the Cue_Type to Pointing - the peer will point to the correct object after eye contact. The agent is trained on the following five problems: Boxes, Switches, Levers, Marble, Generators, and on the asocial version of the Doors problem (a version without the distractor or peer). Training on this asocial version is important as it enables the agent to learn how to use a door, which is needed to evaluate generalization.

5.2 Tole Role reversal experiment

The parameter trees used in this experiment are depicted in figure 15. We used the COLLABORATION type environments. We evaluate agents on role A of the MARBLEPASS task - the agent has to push the marble to the right side of the environment, from where the peer can push it to the *marble generator*.

5.3 The Scaffolding experiment

The parameter trees used in this experiment are depicted in figure 16. In this experiment, we use the INFORMATION SEEKING environment type with the LANGUAGE FEEDBACK cue type. We train agents on all six problems, using different values of the INTRODUCTORY_SEQUENCE and HELP parameters. We evaluate the agents on all six problems, with the most complex introductory sequence - ASK_EYE_CONTACT.

The agent denoted by "scaf_4" is trained on four different values of the INTRODUCTORY SEQUENCE parameter, and with the HELP parameter set to N (the peer will provide cues). This agent will be trained on a total of 18 different environments: six problems, and four introductory sequences. The second agent (denoted by "scaf_8") is also trained on all values of the INTRODUCTORY_SEQUENCE parameter, but it is in addition trained on both values of the HELP parameter (N and Y) - a total of 36 environments. In half of those environments (with HELP set to Y) the peer will provide the apple to the agent after the introduction (e.g. it will go to the correct box, and open it). In the other half (with HELP set to N), the peer will only provide linguistic feedback cues.

6 ADDITIONAL CASE STUDIES

6.1 Inferring the meaning of linguistic cues

In this section, we study the ability of the agent to infer the meaning of simple words. We follow the same procedure as the experiments with the pointing gesture in the main text. This case study is motivated by the experiments from cognitive science, in which the infants' word understanding steadily increased in the period between 9 and 15 months after birth(Carpenter et al., 1998). We study the following questions:

- Can an RL agent learn to interpret simple utterances?
- Can the agent generalize to new situations, and infer the meaning of those utterances for objects in a new context?

The best performing agent on the linguistic environments in the pilot study was the one using the linguistic count-based exploration bonus (PPO-CBL) (see appendix 4). We use this agent to address both questions.

Environments The environments are similar to those in the pointing experiments in the main text: the INFORMATION_SEEKING environment type, with the INTRODUCTORY_SEQUENCE set to EYE_CONTACT. The only difference is that the peer will give linguistic cues instead of pointing. We run two experiments with two different types of linguistic cues: *Color* and *Feedback*. In *Color* the peer will utter name color of the correct object. In *Feedback* the peer will utter a description of how close the agent is to the correct object: "Cold", "Medium", "Warm", and "Hot" meaning, respectively, "far", "medium", "close" and "right next to". As in the pointing experiments, the agent is trained on the five problems and the asocial version of the DOORS problem.

Can RL agents learn to interpret simple utterances? Figure 3 show the performance of the agent with the linguistic count-based exploration bonus (denoted PPO_CBL_train). We can see that the agent (PPO-CBL) solves these environments efficiently, reaching a final performance of 95.9% and 71.4% for

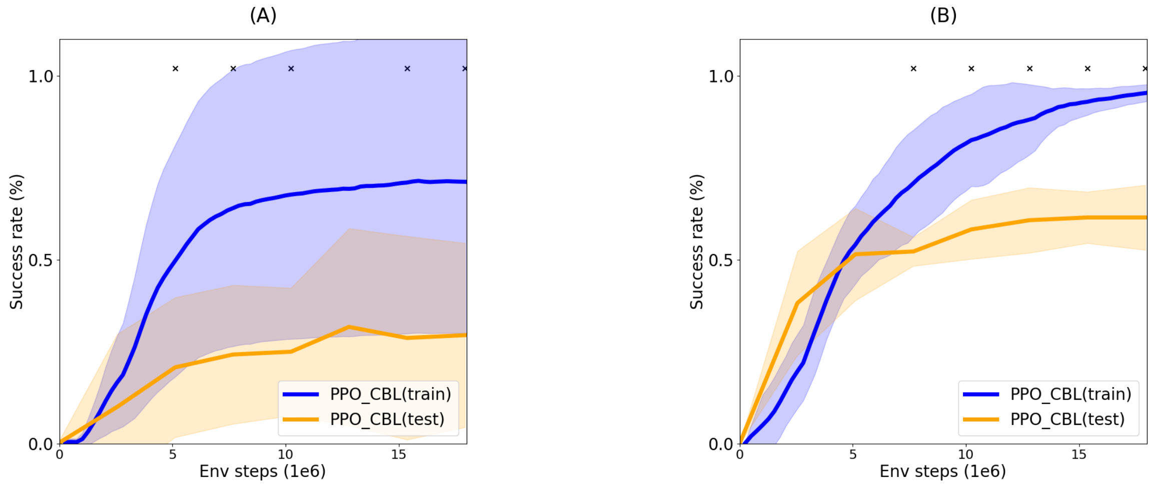

Figure 3. The linguistic cues experiments. We study if an RL agent is able to infer the meaning of linguistic cues in order to use the correct object. We consider two types of cues: *language feedback* and *color*. In both settings, the agent was trained on five different problems, and on the asocial version of the Doors problem (only one door and no peer present in the environment) - denoted by "train". Agents were periodically evaluated on the social version of the Doors problem (two doors and a peer giving cues) - denoted by "test". The figure compares the success rate (mean +/- std over 8 seeds) on the training environments with the evaluation on the testing environment. The cross marks depict statistical significance ($p = 0.05$). In both cases the agents achieve much better performance on the training problems, but fail to generalize to a new problem - the agent is not able to infer the meaning of an utterance in a new context. **(A)** Language Feedback cue type experiments: the peer gives cues regarding the proximity of the agent is to the correct object (e.g. Hot, Warm, Cold). **(B)** Language Color cue type experiments: the peer utters the color of the correct object.

COLOR and FEEDBACK cue types, respectively. We further analyse the performance of each separate seed for the agent trained on the FEEDBACK cue type. This is shown in figure S4 where it is visible that the agent is normally able to achieve high performance, but that there are two seeds which, due to their instability, reach a success rate of 0. This experiment shows that the agent is capable of learning to infer the meaning of simple utterances in familiar contexts.

Can the agent generalize to new situations? A more interesting question is whether that agent can infer the meaning of the same word based on a new context. Therefore, we evaluate the agent's generalization abilities in a new scenario - the DOOR problem. This kind of generalization is particularly interesting as communication depends on our ability to ground words in *new* social contexts: inferring meaning by combining the convention associated to a word with the recursively inferred intention of the speaker. For example, while "red" can mean "open the red box" in one context, it can mean "push the marble towards the red generator" in another.

Figure 3 shows the performance of the same agent evaluated on the DOORS problem (denoted "PPO_CBL_test") They show that neither of the agents is capable of such generalization, which is consistent with the pointing experiments in the main text.

These results motivate future research on what kind of biases could be built into the agents (and in what way) so that they could infer the meaning of familiar words in new contexts. For example, an interesting avenue of future work is to try to combine an agent with a large language models, and see if the knowledge contained in it could make the agent generalize better.

6.2 Joint Attention

Tomasello describes joint attention as consisting of two parts: triangulation and recursiveness (Tomasello, 2019). He argues that joint attention plays a key role in the 9-month revolution by transforming dyadic interactions (e.g. mimicking facial expressions) to triadic (e.g. imitating an action on an object). Joint attention was also required in the experiments with pointing and linguistic cues. The agent and the peer triangulated on an external referent, however, the agent could assume that the peer was participating in the interaction (recursiveness was assumed to be solved).

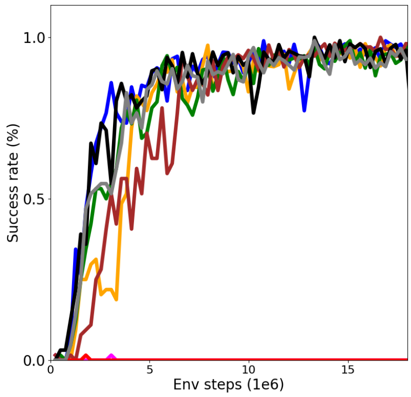

Figure S4. Per-seed performance on the training environments of the agent from figure 3A ("PPO_CBL(train)"). The figure shows that the agent is able to solve the training tasks efficiently, but that there are two unstable seeds which result in the success rate of 0%.

In this experiment, we aim to conduct a more thorough test of the second aspect of joint attention - *recursiveness* (both participants being aware that they are both sharing attention). To solve the task, the agent needs to infer if the peer is participating and is aware that the agent is participating too. We create environments where the peer, in addition to giving regular cues inside joint attention, gives *misleading* cues outside joint attention. These cues are implemented uttering a random cue, and are given before the agent completes the introductory sequence. In other words, the agent should learn to discriminate between cues given for the agent during joint attention (after the introduction) and cues given regardless of the agent outside joint attention (before the introduction). We study the following question:

- Can RL agents learn to differentiate between cues given inside and outside joint attention, i.e. can they learn to infer whether the peer is participating in the interaction?

Environments In this section, we extend the environment from section 6.1 studying the COLOR cue type. The environment is extended so that the agent must also recursively infer whether the cue is intended for the agent. A misleading cue is given before the introductory sequence is completed (the peer utters a color of a random object).

Results Figure S5 compares the performance (success rate) of the agent trained on this extended environment (denoted by JA) with the agent trained on the regular environment (from the experiment in section 6.1). These results show that the agent is not able to differentiate between cues given inside and outside of joint attention. We believe that this is due to the cues being highly misleading in this environment. As the peer utters the color of a random object present in the environment 50% of the time the misleading cue will be the same as the helpful one.

These results open many avenues for future research. One might study which kinds of biases can be integrated into the agent to make such cues less misleading. The generalization abilities of those agents should also be investigated. For instance, we could study if an agent that learned to ignore misleading linguistic cues would ignore misleading pointing cues.

6.3 Imitation learning

In the following section, we study the ability of the agent to learn how to obtain the apple by imitating the peer. This experiment is motivated by an experiment from (Carpenter et al., 1998) where infants showed a steady increase in imitation learning abilities in the period between 9 and 15 months after birth. We want to test the agent's ability to imitate an instrumental action on an new object.

From an AI perspective, this can be seen as meta-imitation learning. We study if an agent can obtain (through gradients) the imitation learning mechanism, which it could then use (during the episode) to learn how to use a new object. We study the following question:

- Can RL agents learn (through gradients) an imitation mechanism to imitate the usage of a new object?

We use the agent with the visual count-based exploration bonus (CB). We compare three agents trained with the same exploration bonus scaled by different weights: 0.25, 0.5, and 1.

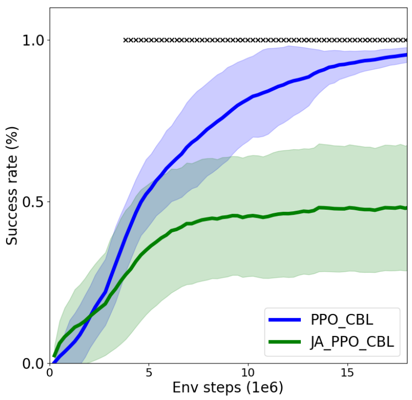

Figure S5. The joint attention experiment. The environments feature a test for recursivness - infer if the peer knows that they are working together. The environments are same as the ones from figure 3B, but with the addition of misleading cues - random cues given regardless of the agent (a random color). The peer gives misleading cues outside of joint attention (before the introductory sequence). The agent should ignore these cues, and use only cues given inside joint attention. The figure compares the success rate (mean +- std over 8 seeds) of the agent trained on the environments with both regular and misleading cues("JA_PPO_CBL"), to the agent trained on the environments with only regular cues ("PPO_CBL(train)" from figure 3B). The figure shows that the agent is unable to master the Joint Attention variant.

Environment The Environment is an INFORMATION SEEKING type environment without a distractor. After the introductory sequence (EYE_CONTACT), the peer will demonstrate using an object to obtain the apple. For example, it will toggle a box or push a generator. Then the peer will then eat the apple, and revert the environment to its initial state. The agent should then imitate the peer - use the same action on the object - to obtain the apple for itself. If the agent uses the object it in the wrong way (e.g. pushes the box instead of toggling it) it will be blocked and the apple will not be obtainable in this episode. The agents are evaluated on a new problem in which the agent encounters a new object for the first time. This means that the agent must pay attention to how the peer uses the object, and use it in the same way. [2]

The agents are trained on five problems (all expect DOORS). Most importantly, compared to the experiments with pointing and linguistic cues, these agents will not be trained on the asocial version of the DOORS problem. That is because, in the generalization testing, we test if the agent can learn to use a completely new object.

Results Figure 6 shows the performance (success rate) of the agents on the training environments, the percentage of succesful introduction with the peer, and the evaluation on the (unseen) DOORS problem. Figures 6A and 6B show that the agent with a lot of exploration bonus (PPO_CB_1) is too focused on the peer and, and is unable to solve the task. This is implied by the high percentage of the successful

[2] The encoding of the peer includes the peer's previous timestep action.

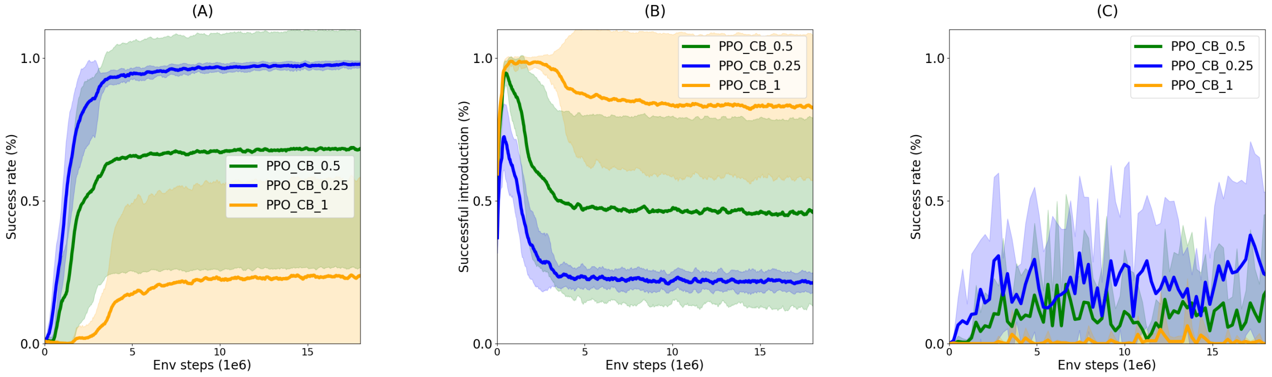

Figure 6. Imitation learning experiments. The peer demonstrates how to use an object (after the agent successfully introduces itself). The agent is trained on five different problems and evaluated on a new problem with a previously unobserved object (a door). A socially proficient agent should be able to learn (by observing the demonstration) which action (toggle or push) to use on the new object. The curves compare three agents trained with a different scaling factor for the visual count-based exploration bonus. One can see that the agent with high exploration bonus ("PPO_CB_1") focuses too much on the peer, which results in ignoring the task. This is evidenced by high success in completing the introductory sequence (B), but low success rate on the task (A). On the other hand, using low exploration bonus ("PPO_CB_0.25") pushes the agent to solve the training task whilst ignoring the peer. Rather than observing the peer's demonstration, this agent learns how to use objects by themselves. This results in perfect performance on the training object, but it makes it impossible to generalize to a new object. Neither of the agents is able to achieve high performance on the heldout testing environment. This implies that they are not able to learn (online) through imitation which action to use with a new object. **(A)** Imitation experiments performance (success rate) on the training environments. **(B)** The percentage of successful introductory sequences on the training environments. **(C)** Imitation experiments performance (success rate) on the testing environment.

introductory sequence, and low success rate on the training environments. On the other hand the agent with smaller exploration bonus weight (PPO_CB_0.25) solves these environments without problems, however it does use the peer. As such the agent can solve the training environments by ignoring the peer and discovering how to use each object by itself. However, this agent is not able to generalize to a new object as the only way to know how to use that object is to observe the peer's demonstration (see figure 6C). Figure 6C shows the performance of those agents on the testing environment. The figure shows that neither of the three agents is capable of acquiring an meta-imitation learning mechanism that can generalize to a novel object.

These results are not surprising, as current exploration bonuses are not well suited to enable RL agents to meta-learn mechanisms. These results imply that an interesting avenue of research is to study how to endow agents with such meta-imitation learning mechanisms that would enable them to learn a behavior in a new scenario. An promising solution to this problem are large language models and other large transformer-based networks pretrained on many other tasks. It would be interesting to study if such agents already have an imitation learning mechanism which would enable such online imitation. This would open up countless avenues of research into various forms of online imitation and emulation learning.

6.4 Inferring another's field of view

In this section, we study the ability of the agent to infer what the other observes. This experiment is modeling the one in Hare et al. (2001). In it, apes were shown to be able to infer what another sees, as they only took the food the alpha male could not see. We study the following question:

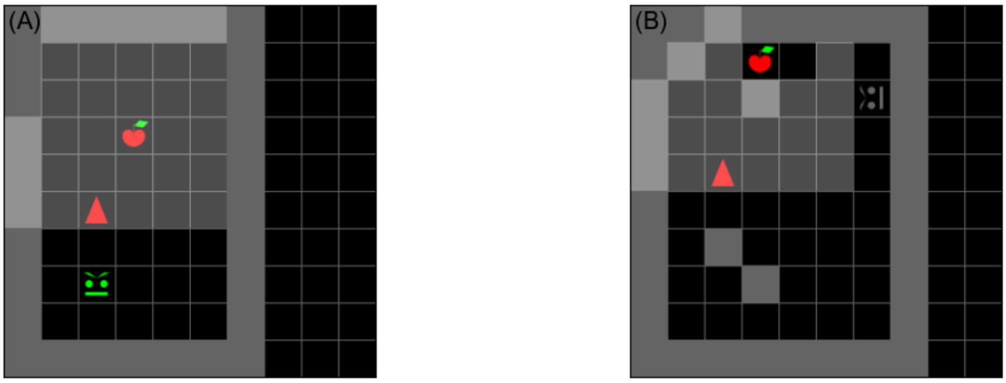

Figure 7. Environments from the Adversarial peer experiments in which the agent has to infer the peer's field of view. The agent is rewarded upon eating the apple on the condition that it was not in the field of view of the peer while doing so. We run the experiments with two different settings: with and without occlusions. Occlusions make it harder to infer the peer's field of view as it is no longer rectangular. **(A)** Adversarial peer environment without occlusions. **(B)** Adversarial peer environment with occlusions.

- Can agents learn to infer the other's field of view?

Environment We use the AdversarialPeer environment type, in which the agent has to eat the apple while not being seen by the peer. We study two versions of this environment: with and without obstacles (for more details, refer to appendix 3). Obstacles make the problem of inferring the peer's field of view harder.

Experiment We study how the agent infers the peer's field of view by training the agent on the AdversarialPeer task. It is important to note that this agent can sometimes use other (asocial) information to achieve performance. For example, if the object is surrounded by occlusions the agent could guess that it is not observed by the peer, which is not necessarily the case. To better understand the performance of the agent we compare the agent with two baselines. First, we assess to what extent the agent is making inferences based on the peer's location and gaze direction. We train an agent ("invisible_peer") that has the peer filtered from the its observations (it cannot observe the peer). This baselines estimates the maximum possible performance If the standard agent outperforms this baseline this implies that it is leveraging the social information in the environment. Second, to estimate the upper bound on the performance we train an agent in the environment without the peer present (this agent is reward every time it eats the apple).

Results Figure 8 shows the performances of those three agents. It shows that the agent outperforms the agent with the peer filtered from its observations ("invisible_peer"), which implies that the agent is using the peer's location and gaze direction to infer weather to eat the apple or not. Furthermore, the agent is not able to match performance of the agent trained without the peer present in the environment ("no_peer"). This results imply that, while the agent is able to leverage some social information in the environment, there still remains room for improvement. Future research could focus on constructing novel types of exploration bonuses to bridge this gap.

6.5 Formats

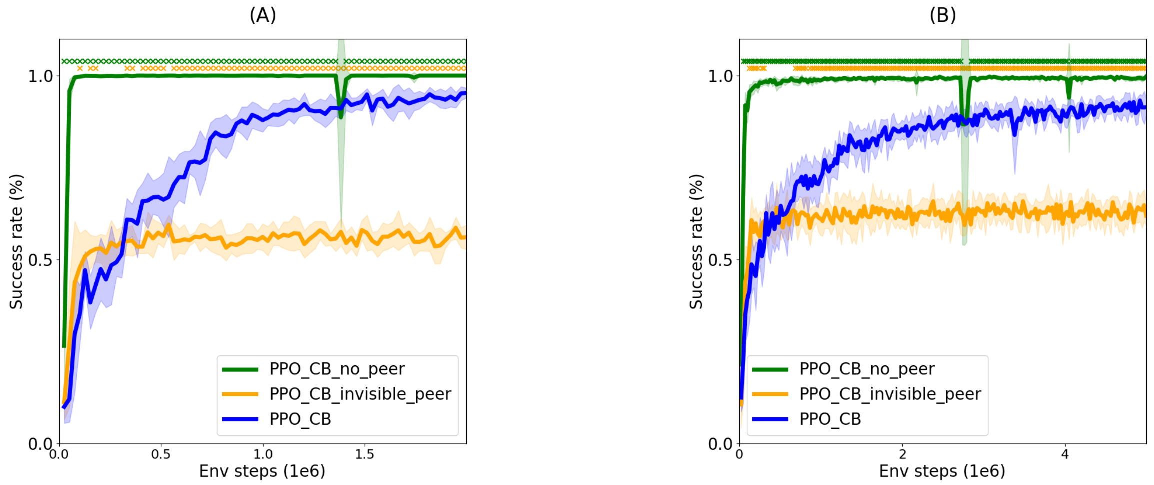

Figure 8. Adversarial peer experiments. We compare three agents on two environments (depicted on figure 7). The "PPO_CB" agent is trained on the regular environment (rewarded upon eating the apple while not being observed by the peer). The "PPO_CB_no_peer" agent is trained in the environment without the peer (the agent is rewarded every time it eats the apple). This represents the upper bound of the performance. The "PPO_CB_invisible_peer" agent is trained on the regular environment with the peer filtered from the agent's observations. This represents the performance of a completely asocial agent which ignores the peer. Figure compares the performance of these three agents (8 seeds +- std), the crosses depict a statistically significant difference (p¡0.05) compared to the "PPO_CB" agent. The results show that the "PPO_CB" agent is able to partially infer the peer's field of view (as it outperforms the "invisible_peer" baseline), but is not able to reach perfect performance (as defined by the "PPO_CB_no_peer" baseline). **(A)** No occlusions. **(B)** Occlusions

In the following experiment, we study the ability of the agent to learn formats (also referred to as pragmatic frames in (Vollmer et al., 2016)). Formats are a concept introduced by Jerome Bruner. They can be regarded as protocols of social interactions. We study the following question:

- To what extend can an exploration bonus help with the acquisition of a complex format.

We address this question by training two agents (one with an exploration bonus, and one without it).

Environment We use the INFORMATION SEEKING environment type with the LANGUAGE FEEDBACK cue type. We train all agents on all six problems. In contrast to section 6.1, where the introductory sequence was always set to EYE CONTACT, here it is set to ASK EYE CONTACT - the peer will give cues after the agent utters "Help, please" during eye contact.

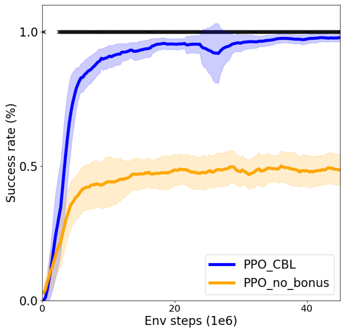

Figure S9. Comparison of an agent with and without the exploration bonus on an environment with a more complex introductory sequence (format). The task consists of the agent doing the introductory sequence by making eye contact and uttering "Help, please". The peer will then give linguistic cues regarding the proximity of the agent to the target object (e.g. Hot, Warm, Cold). Based on these cues, the agent should use the target object, instead of the distractor, to obtain the apple. The figure shows that using the visual count-based exploration bonus enables the agent to learn a more complex introductory sequence and solve the task.

Table S1. Template-based grammar used in all of the SocialAI environments. If the agent decided to speak it chooses a template and a noun to insert into the template.

**Nouns**		
Action	Template	Noun
0	Where is <noun>	please
1	Help <noun>	the exit
2	Close <noun>	the wall
3	How are <noun>	you
4		the ceiling
5		the window
6		the entrance
7		the closet
8		the drawer
9		the fridge
10		the floor
11		the lamp
12		the trash can
13		the chair
14		the bed
15		the sofa

Table S2. Examples of actions in the environment. Second and third dimension must both either be undefined or not. In practice, there is an additional binary output which defines if the agent will speak.

Action	description
(1, -, -)	moves left without speaking
(1, 1, 5)	moves left and utters ”Help the window”
(-, 1, 5)	doesn’t move but utters ”Help the window”
(-, -, -)	nothing happens

Results Figure S9 compares the performance of an agent that does not use any exploration bonus (”PPO_no_bonus”) to an agent that uses the visual count-based exploration bonus (”PPO_CBL”). The agent with the exploration bonus achieves high performance (97.9% success rate) and greatly outperforms the agent without the exploration bonus. These experiments show that, as expected, learning complex formats can be made easier with exploration bonuses.

This experiment can be interpreted in tandem with the experiment on scaffolding in the main text. In that experiment, we showed how more complex formats can be learned by weaker agents (without an exploration bonus) when learning in a scaffolded environment. Future work could explore how these two different approaches - modifying the agent and modifying the environment - can be used in tandem to learn even more complex formats. Furthermore, one interesting research direction is to study which kinds of problems are better addressed by modifying the agent and which by modifying the environment.

6.6 Additional information on the case study with large language models as interactive agents

Figures S11, S12, and S13 show the in-context examples provided to large language models in the experiments in the main text for the AsocialBox environment, ColorBoxes environment, and the generalization to the ColorBoxes environment, respectively. The in-context examples were created by hand.

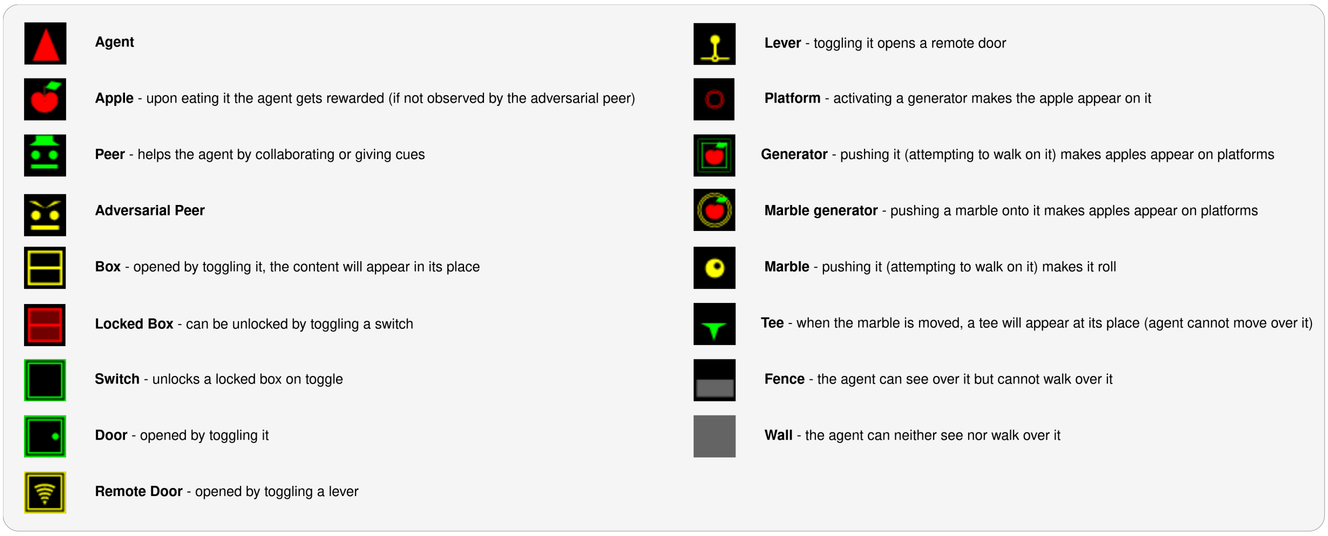

Figure S10. Visualizations and descriptions for all objects featured in SocialAI environments.

REFERENCES

Burda, Y., Edwards, H., Storkey, A. J., and Klimov, O. (2018). Exploration by random network distillation. *CoRR* abs/1810.12894

Carpenter, M., Nagell, K., and Tomasello, M. (1998). Social cognition, joint attention, and communicative competence from 9 to 15 months of age. *Monographs of the Society for Research in Child Development* 63 4, i–vi, 1–143

Chevalier-Boisvert, M., Bahdanau, D., Lahlou, S., Willems, L., Saharia, C., Nguyen, T. H., et al. (2019). Babyai: A platform to study the sample efficiency of grounded language learning. In *7th International Conference on Learning Representations, ICLR 2019, New Orleans, LA, USA, May 6-9, 2019* (OpenReview.net)

Chung, J., Gülçehre, Ç., Cho, K., and Bengio, Y. (2015). Gated feedback recurrent neural networks. In *Proceedings of the 32nd International Conference on Machine Learning, ICML 2015, Lille, France, 6-11 July 2015*, eds. F. R. Bach and D. M. Blei (JMLR.org), vol. 37 of *JMLR Workshop and Conference Proceedings*, 2067–2075

Hare, B., Call, J., and Tomasello, M. (2001). Do chimpanzees know what conspecifics know? *Animal behaviour* 61, 139–151

Hochreiter, S. and Schmidhuber, J. (1997). Long short-term memory. *Neural Comput.* 9, 1735–1780. doi:10.1162/neco.1997.9.8.1735

[Dataset] Hui, D. Y.-T., Chevalier-Boisvert, M., Bahdanau, D., and Bengio, Y. (2020). Babyai 1.1

Krizhevsky, A., Sutskever, I., and Hinton, G. E. (2012). Imagenet classification with deep convolutional neural networks. *Advances in neural information processing systems* 25, 1097–1105

LeCun, Y., Boser, B., Denker, J. S., Henderson, D., Howard, R. E., Hubbard, W., et al. (1989). Backpropagation applied to handwritten zip code recognition. *Neural computation* 1, 541–551

Pathak, D., Agrawal, P., Efros, A. A., and Darrell, T. (2017). Curiosity-driven exploration by self-supervised prediction. In *ICML*

Perez, E., Strub, F., de Vries, H., Dumoulin, V., and Courville, A. C. (2017). Film: Visual reasoning with a general conditioning layer. *CoRR* abs/1709.07871

Raileanu, R. and Rocktäschel, T. (2020). RIDE: rewarding impact-driven exploration for procedurally-generated environments. *CoRR* abs/2002.12292

```
New episode.
Obs : 1 steps in front of you and 1 steps to the left there is a
      closed green lockablebox
Act : move forward
Obs : Just to the left of you there is a closed green lockablebox
Act : turn left
Obs : Right in front of you there is a closed green lockablebox
Act : toggle
Obs : Right in front of you there is a red apple
Act : toggle
Obs : Right in front of you there is a yellow apple
Success!
New episode.
Obs : 1 steps in front of you and 1 steps to the right there is a
      closed blue lockablebox
Act : move forward
Obs : Just to the right of you there is a closed blue lockablebox
Act : turn right
Obs : Right in front of you there is a closed blue lockablebox
Act : toggle
Obs : Right in front of you there is a red apple
Act : toggle
Obs : Right in front of you there is a yellow apple
Success!
New episode.
Obs : 3 steps in front of you there is a closed green lockablebox
Act : move forward
Obs : 2 steps in front of you there is a closed green lockablebox
Act : move forward
Obs : Right in front of you there is a closed green lockablebox
Act : toggle
Obs : Right in front of you there is a red apple
Act : toggle
Obs : Right in front of you there is a yellow apple
Success!
New episode.
Obs : 3 steps in front of you and 1 steps to the left there is a
      closed brown lockablebox
Act : move forward
Obs : 2 steps in front of you and 1 steps to the left there is a
      closed brown lockablebox
Act : move forward
Obs : 1 steps in front of you and 1 steps to the left there is a
      closed brown lockablebox
Act : move forward
Obs : Just to the left of you there is a closed brown lockablebox
Act : turn left
Obs : Right in front of you there is a closed brown lockablebox
Act : toggle
Obs : Right in front of you there is a red apple
Act : toggle
Obs : Right in front of you there is a yellow apple
Success!
```

```
New episode.
Obs : 2 steps in front of you and 3 steps to the right there is a
      closed blue lockablebox
Act : move forward
Obs : 1 steps in front of you and 3 steps to the right there is a
      closed blue lockablebox
Act : move forward
Obs : 3 steps to the right there is a closed blue lockablebox
Act : turn right
Obs : 3 steps in front of you there is a closed blue lockablebox
Act : move forward
Obs : 2 steps in front of you there is a closed blue lockablebox
Act : move forward
Obs : Right in front of you there is a closed blue lockablebox
Act : toggle
Obs : Right in front of you there is a red apple
Act : toggle
Obs : Right in front of you there is a yellow apple
Success!
New episode.
Obs :
Act : turn right
Obs :
Act : turn right
Obs : 1 steps in front of you and 1 steps to the right there is a
      closed brown lockablebox
Act : move forward
Obs : Just to the right of you there is a closed brown lockablebox
Act : turn right
Obs : Right in front of you there is a closed brown lockablebox
Act : toggle
Obs : Right in front of you there is a red apple
Act : toggle
Obs : Right in front of you there is a yellow apple
Success!
```

Figure S11. In context examples for the ASocialBox environment.

Savinov, N., Raichuk, A., Marinier, R., Vincent, D., Pollefeys, M., Lillicrap, T. P., et al. (2018). Episodic curiosity through reachability. *ArXiv* abs/1810.02274

Schulman, J., Wolski, F., Dhariwal, P., Radford, A., and Klimov, O. (2017). Proximal policy optimization algorithms. *ArXiv* abs/1707.06347

[Dataset] Tang, H., Houthooft, R., Foote, D., Stooke, A., Chen, X., Duan, Y., et al. (2017). Exploration: A study of count-based exploration for deep reinforcement learning

Tomasello, M. (2019). Becoming human. In *Becoming Human* (Harvard University Press)

Vollmer, A.-L., Wrede, B., Rohlfing, K. J., and Oudeyer, P.-Y. (2016). Pragmatic frames for teaching and learning in human–robot interaction: Review and challenges. *Frontiers in Neurorobotics* 10, 10. doi:10.3389/fnbot.2016.00010

```
New episode.
Obs : 1 steps in front of you and 2 steps to the left there is a
      closed brown lockablebox
Caretaker says: blue
Act : turn left
Obs : 3 steps in front of you and 3 steps to the left there is a
      caretaker
1 steps in front of you and 1 steps to the left there is a closed
      blue lockablebox
2 steps in front of you and 1 steps to the right there is a closed
      brown lockablebox
Caretaker says: blue
Act : move forward
Obs : 2 steps in front of you and 3 steps to the left there is a
      caretaker
Just to the left of you there is a closed blue lockablebox
1 steps in front of you and 1 steps to the right there is a closed
      brown lockablebox
Caretaker says: blue
Act : turn left
Obs : Right in front of you there is a closed blue lockablebox
3 steps in front of you and 2 steps to the right there is a
      caretaker
Caretaker says: blue
Act : toggle
Obs : Right in front of you there is a red apple
3 steps in front of you and 2 steps to the right there is a
      caretaker
Caretaker says: blue
Act : toggle
Obs : Right in front of you there is a yellow apple
3 steps in front of you and 2 steps to the right there is a
      caretaker
Caretaker says: blue
Success!
New episode.
Obs : 3 steps in front of you there is a caretaker
Act : turn left
Obs : 2 steps to the left there is a closed blue lockablebox
5 steps in front of you there is a closed green lockablebox
 3 steps to the right there is a caretaker
Caretaker says: blue
Act : turn left
Obs : 2 steps in front of you there is a closed blue lockablebox
Caretaker says: blue
Act : move forward
Obs : Right in front of you there is a closed blue lockablebox
Caretaker says: blue
Act : toggle
Obs : Right in front of you there is a red apple
Caretaker says: blue
Act : toggle
Obs : Right in front of you there is a yellow apple
Caretaker says: blue
Success!
New episode.
Obs : 2 steps in front of you and 2 steps to the left there is a
      closed blue lockablebox
Caretaker says: brown
Act : turn left
Obs : 3 steps in front of you and 1 steps to the left there is a
      caretaker
2 steps in front of you and 2 steps to the right there is a closed
      blue lockablebox
Caretaker says: brown
Act : turn left
Obs : 4 steps in front of you there is a closed brown lockablebox
1 steps in front of you and 3 steps to the right there is a
      caretaker
Caretaker says: brown
Act : move forward
Obs : 3 steps in front of you there is a closed brown lockablebox
 3 steps to the right there is a caretaker
Caretaker says: brown
Act : move forward
Obs : 2 steps in front of you there is a closed brown lockablebox
Caretaker says: brown
Act : move forward
Obs : Right in front of you there is a closed brown lockablebox
Caretaker says: brown
Act : toggle
Obs : Right in front of you there is a red apple
Caretaker says: brown
Act : toggle
Obs : Right in front of you there is a yellow apple
Caretaker says: brown
Success!
New episode.
Obs : Just to the right of you there is a closed green lockablebox
Caretaker says: brown
Act : turn left
Obs : 4 steps in front of you and 3 steps to the right there is a
      closed brown lockablebox
Caretaker says: brown
Act : move forward
Obs : 3 steps in front of you and 3 steps to the right there is a
      closed brown lockablebox
Caretaker says: brown
Act : move forward
Obs : 2 steps in front of you and 3 steps to the right there is a
      closed brown lockablebox
Caretaker says: brown
Act : move forward
Obs : 1 steps in front of you and 3 steps to the right there is a
      closed brown lockablebox
Caretaker says: brown
Act : move forward
Obs : 3 steps to the right there is a closed brown lockablebox
Caretaker says: brown
Act : turn right
Obs : 5 steps in front of you there is a caretaker
3 steps in front of you there is a closed brown lockablebox
Caretaker says: brown
Act : move forward
Obs : 4 steps in front of you there is a caretaker
2 steps in front of you there is a closed brown lockablebox
Caretaker says: brown
Act : move forward
Obs : 3 steps in front of you there is a caretaker
Right in front of you there is a closed brown lockablebox
Caretaker says: brown
Act : toggle
Obs : 3 steps in front of you there is a caretaker
Right in front of you there is a red apple
Caretaker says: brown
Act : toggle
Obs : 3 steps in front of you there is a caretaker
Right in front of you there is a yellow apple
Caretaker says: brown
Success!
New episode.
Obs :
Caretaker says: blue
Act : turn left
Obs : 3 steps to the left there is a caretaker
Caretaker says: blue
Act : turn left
Obs : 1 steps in front of you and 1 steps to the left there is a
      closed blue lockablebox
3 steps in front of you there is a caretaker
4 steps in front of you and 2 steps to the right there is a closed
      brown lockablebox
Caretaker says: blue
Act : move forward
Obs : Just to the left of you there is a closed blue lockablebox
2 steps in front of you there is a caretaker
3 steps in front of you and 2 steps to the right there is a closed
      brown lockablebox
Caretaker says: blue
Act : turn left
Obs : Right in front of you there is a closed blue lockablebox
 2 steps to the right there is a caretaker
Caretaker says: blue
Act : toggle
Obs : Right in front of you there is a red apple
 2 steps to the right there is a caretaker
Caretaker says: blue
Act : toggle
Obs : Right in front of you there is a yellow apple
 2 steps to the right there is a caretaker
Caretaker says: blue
Success!
```

Figure S12. In context examples for the ColorBoxes environment.

```
New episode.
Obs : 2 steps in front of you and 1 steps to the left there is a
      unactivated green lever
2 steps in front of you and 1 steps to the right there is a
      unactivated blue lever
Caretaker says: green
Act : move forward
Obs : 1 steps in front of you and 1 steps to the left there is a
      unactivated green lever
1 steps in front of you and 1 steps to the right there is a
      unactivated blue lever
Caretaker says: green
Act : move forward
Obs : Just to the left of you there is a unactivated green lever
Just to the right of you there is a unactivated blue lever
Caretaker says: green
Act : turn left
Obs : Right in front of you there is a unactivated green lever
Caretaker says: green
Act : toggle
Obs : Right in front of you there is a activated green lever
Caretaker says: green
Act : turn left
Obs : 6 steps in front of you and 2 steps to the left there is a
      open green remotedoor
Just to the left of you there is a unactivated blue lever
Just to the right of you there is a activated green lever
5 steps in front of you and 2 steps to the right there is a
      caretaker
Caretaker says: green
Act : move forward
Obs : 5 steps in front of you and 2 steps to the left there is a
      open green remotedoor
4 steps in front of you and 2 steps to the right there is a
      caretaker
Caretaker says: green
Act : turn left
Obs : 1 steps in front of you and 1 steps to the left there is a
      unactivated blue lever
Caretaker says: green
Act : move forward
Obs : Just to the left of you there is a unactivated blue lever
Caretaker says: green
Act : move forward
Obs :
Caretaker says: green
Act : turn right
Obs : 6 steps in front of you there is a red apple
5 steps in front of you there is a open green remotedoor
Caretaker says: green
Act : move forward
Obs : 5 steps in front of you there is a red apple
4 steps in front of you there is a open green remotedoor
Caretaker says: green
Act : move forward
Obs : 4 steps in front of you there is a red apple
3 steps in front of you there is a open green remotedoor
Caretaker says: green
Act : move forward
Obs : 3 steps in front of you there is a red apple
2 steps in front of you there is a open green remotedoor
Caretaker says: green
Act : move forward
Obs : 2 steps in front of you there is a red apple
Right in front of you there is a open green remotedoor
Caretaker says: green
Act : move forward
Obs : Right in front of you there is a red apple
Caretaker says: green
Act : toggle
Obs : Right in front of you there is a yellow apple
Caretaker says: green
Success!
New episode.
Obs : 2 steps in front of you and 1 steps to the right there is a
      closed green lockablebox
Act : move forward
Obs : 1 steps in front of you and 1 steps to the right there is a
      closed green lockablebox
Act : move forward
Obs : Just to the right of you there is a closed green lockablebox
Act : turn right
Obs : Right in front of you there is a closed green lockablebox
Act : toggle
Obs : Right in front of you there is a red apple
...
...
...
...
...
...
Obs : 2 steps in front of you there is a red apple
2 steps in front of you and 1 steps to the right there is a round
      green applegenerator
Just to the right of you there is a green marble
 2 steps to the right there is a round brown applegenerator
Caretaker says: brown
Act : move forward
Obs : Right in front of you there is a red apple
1 steps in front of you and 1 steps to the right there is a round
      green applegenerator
Caretaker says: brown
Act : toggle
Obs : Right in front of you there is a yellow apple
1 steps in front of you and 1 steps to the right there is a round
      green applegenerator
Caretaker says: brown
Success!
New episode.
Obs : 2 steps in front of you and 2 steps to the left there is a
      locked green lockablebox
 2 steps to the left there is a green switch
5 steps in front of you and 3 steps to the right there is a
      caretaker
Caretaker says: green
Act : turn left
Obs : 1 steps in front of you and 1 steps to the left there is a
      blue switch
2 steps in front of you there is a green switch
2 steps in front of you and 2 steps to the right there is a locked
      green lockablebox
Caretaker says: green
Act : move forward
Obs : Just to the left of you there is a blue switch
Right in front of you there is a green switch
1 steps in front of you and 2 steps to the right there is a locked
      green lockablebox
Caretaker says: green
Act : toggle
Obs : Just to the left of you there is a blue switch
Right in front of you there is a green switch
1 steps in front of you and 2 steps to the right there is a closed
      green lockablebox
Caretaker says: green
Act : turn right
Obs : 2 steps in front of you and 1 steps to the left there is a
      closed green lockablebox
Just to the left of you there is a green switch
Caretaker says: green
Act : move forward
Obs : 1 steps in front of you and 1 steps to the left there is a
      closed green lockablebox
Caretaker says: green
Act : move forward
Obs : Just to the left of you there is a closed green lockablebox
Caretaker says: green
Act : turn left
Obs : 3 steps to the left there is a blue switch
1 steps in front of you and 2 steps to the left there is a green
      switch
Right in front of you there is a closed green lockablebox
Caretaker says: green
Act : toggle
Obs : 3 steps to the left there is a blue switch
1 steps in front of you and 2 steps to the left there is a green
      switch
Right in front of you there is a red apple
Caretaker says: green
Act : toggle
Obs : 3 steps to the left there is a blue switch
1 steps in front of you and 2 steps to the left there is a green
      switch
Right in front of you there is a yellow apple
Caretaker says: green
Success!
```

Figure S13. In context examples for the ColorBoxes environment when tested for generalization.

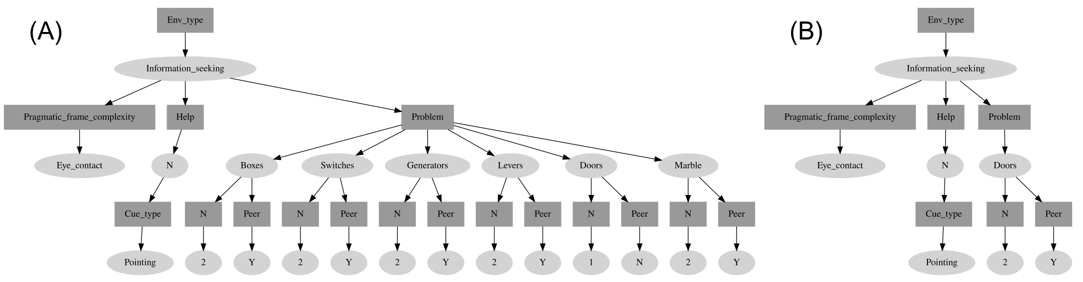

Figure 14. Sampling trees used in the pointing case study in the main text. **(A)** Training sampling tree **(B)** Testing sampling tree - Social Doors

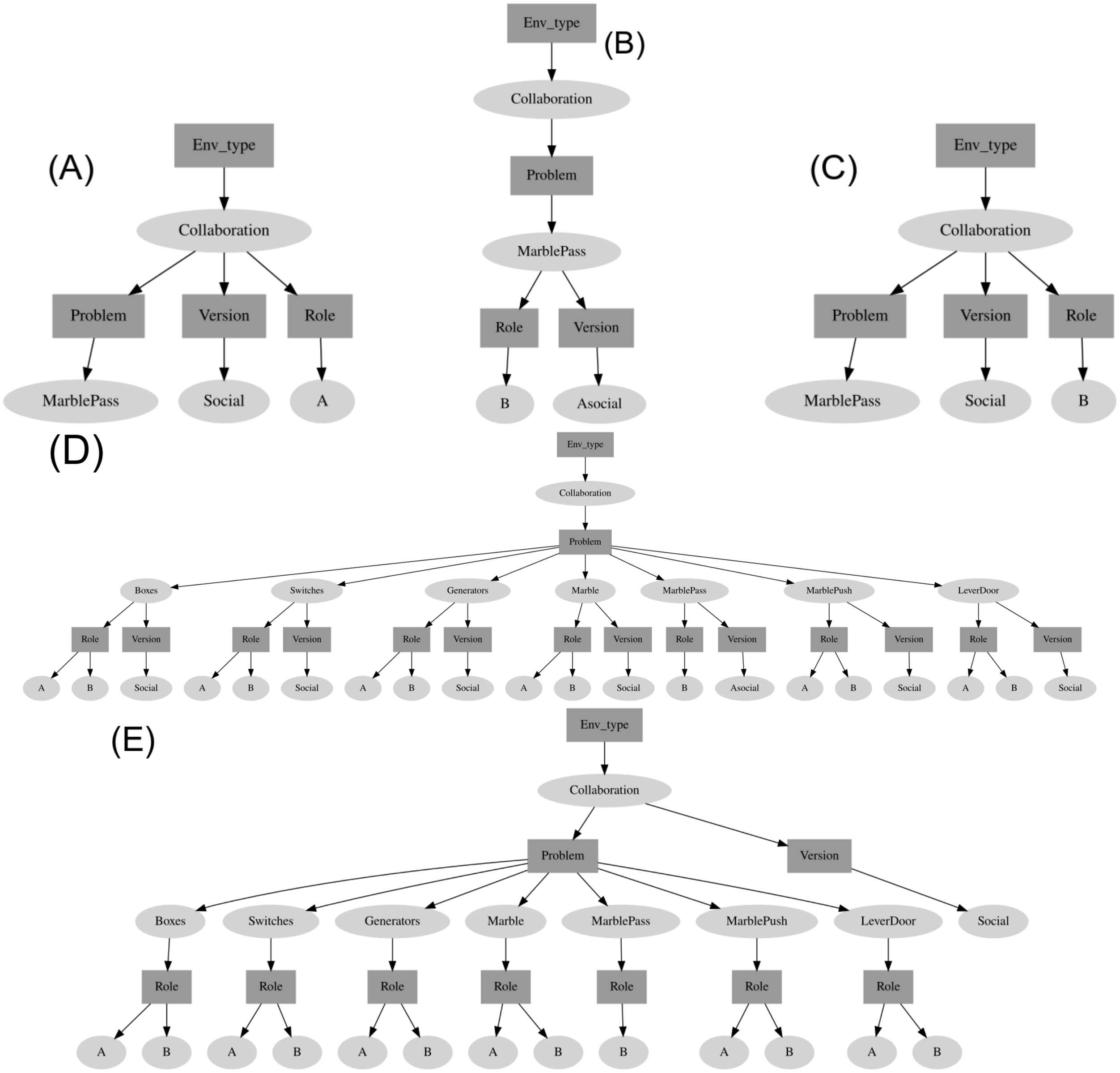

Figure 15. Role reversal sampling trees from the case study in the main text. **(A)** Role A **(B)** Asocial single setting **(C)** Role B single setting **(D)** Asocial group setting **(E)** Role B group setting

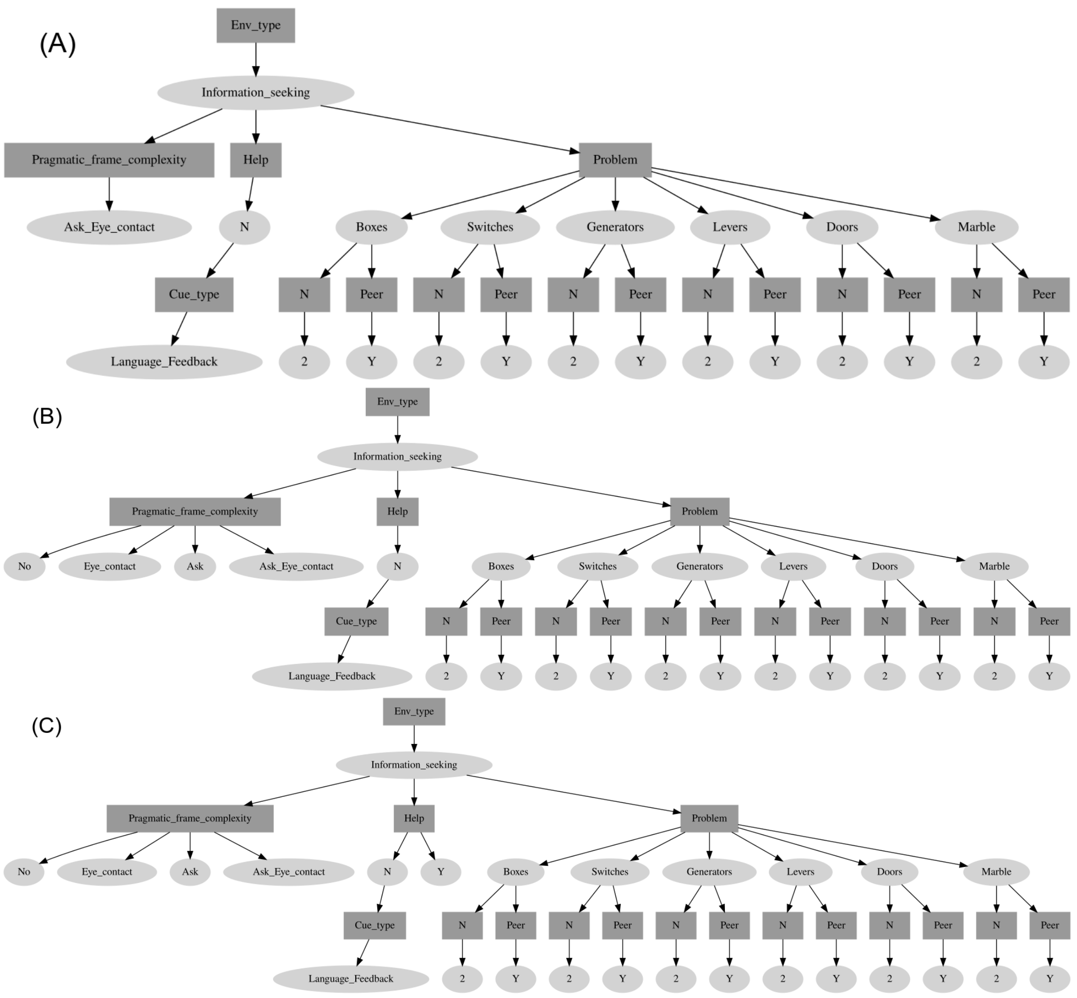

Figure 16. Sampling trees used in the first phase of the scaffolding case study in **(A)** Testing tree. **(B)** Scaf_4 tree. **(C)** Scaf_8 tree.

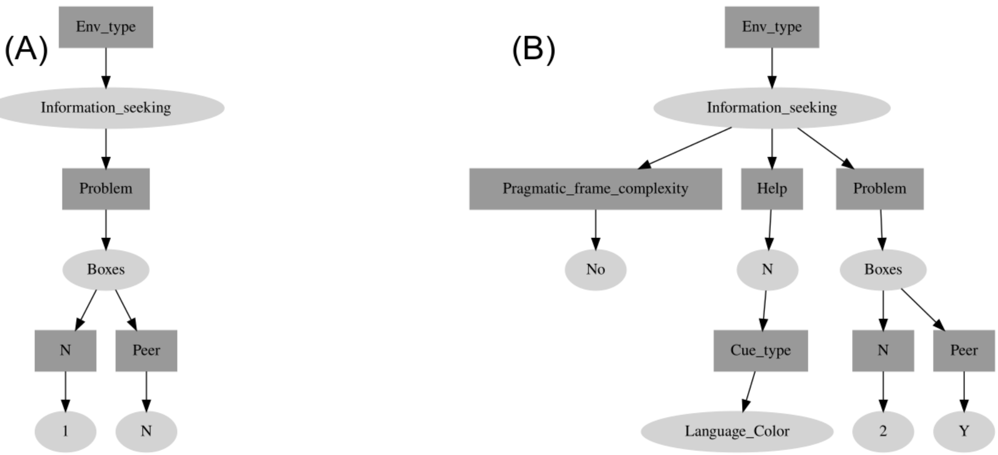

Figure 17. Sampling trees used for evaluation in the experiments with LLM-based interactive agents. **(A)** Asocial Apple **(B)** Color boxes
